# Supplementary material for: Lack of association between early on-treatment HBeAg seroclearance and development of hepatocellular carcinoma or decompensated cirrhosis
Source: JHEP Rep. 2024 Apr 8;6(7):101089. doi: 10.1016/j.jhepr.2024.101089 (PMC11225842; doi:10.1016/j.jhepr.2024.101089)
Supplement: Multimedia component 1 [file mmc1.pdf]

Lack of association between early on-treatment HBeAg seroclearance and development of hepatocellular carcinoma or decompensated cirrhosis

Hyunjae Shin, Won-Mook Choi, Seung Up Kim, Yunmi Ko, Youngsu Park, Jeayeon Park, Moon Haeng Hur, Min Kyung Park, Yun Bin Lee, Yoon Jun Kim, Jung-Hwan Yoon, Jeong-Hoon Lee, Fabien Zoulim

Table of contents

Fig. S1……………………………………………………………………………2
Fig. S2……………………………………………………………………………3
Fig. S3……………………………………………………………………………4
Fig. S4……………………………………………………………………………5
Fig. S5……………………………………………………………………………6
Fig. S6……………………………………………………………………………7
Fig. S7……………………………………………………………………………8
Table S1…………………………………………………………………………..9
Table S2…………………………………………………………………………10
Table S3…………………………………………………………………………11
Table S4…………………………………………………………………………12
Table S5…………………………………………………………………………13
Table S6…………………………………………………………………………14
Table S7…………………………………………………………………………15
Table S8…………………………………………………………………………16
Table S9……………………………………………………………………......17

Supplementary Figures

Fig. S1. Cumulative incidence rate of HBeAg-seroclearance.

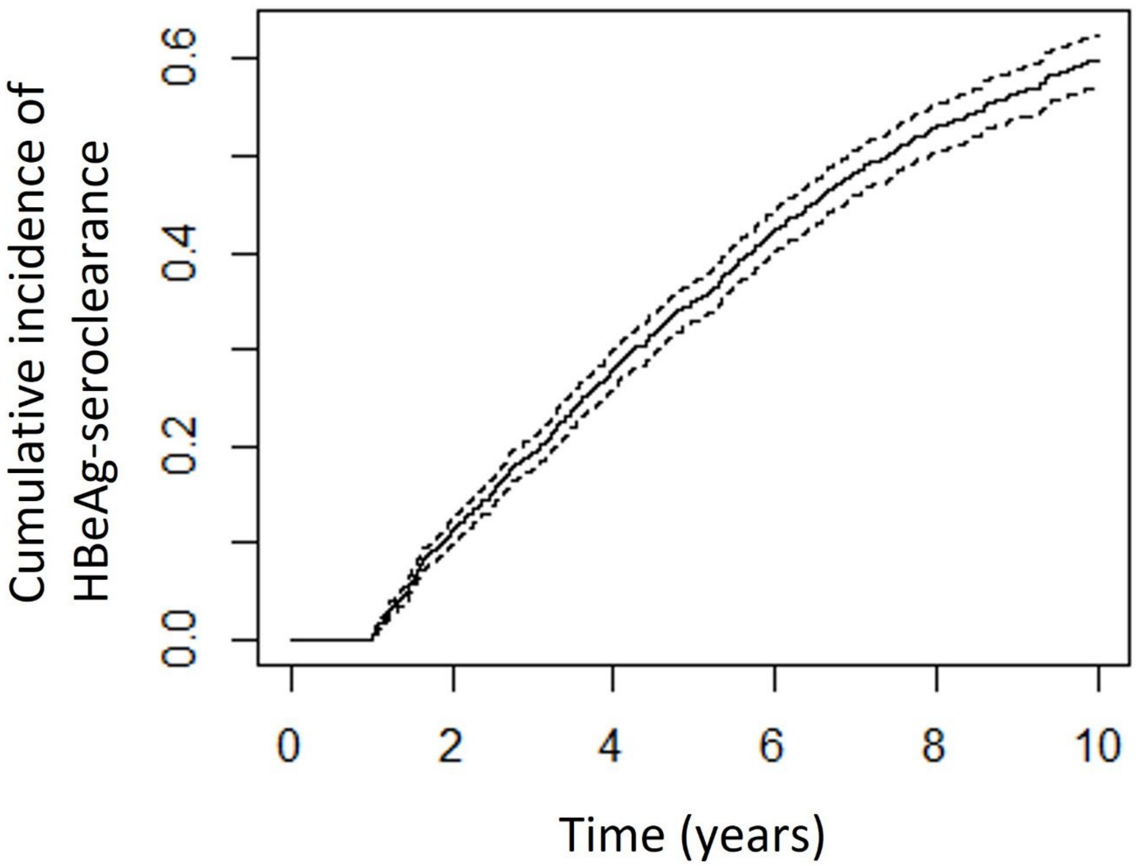

HBeAg, hepatitis B virus envelope antigen

Fig. S2. Risk of (A) decompensated liver cirrhosis, and (B) liver-related outcomes according to HBeAg-seroclearance at 36 months after initiation of antiviral treatment. Weight analysis balanced by inverse probability of treatment weighting.

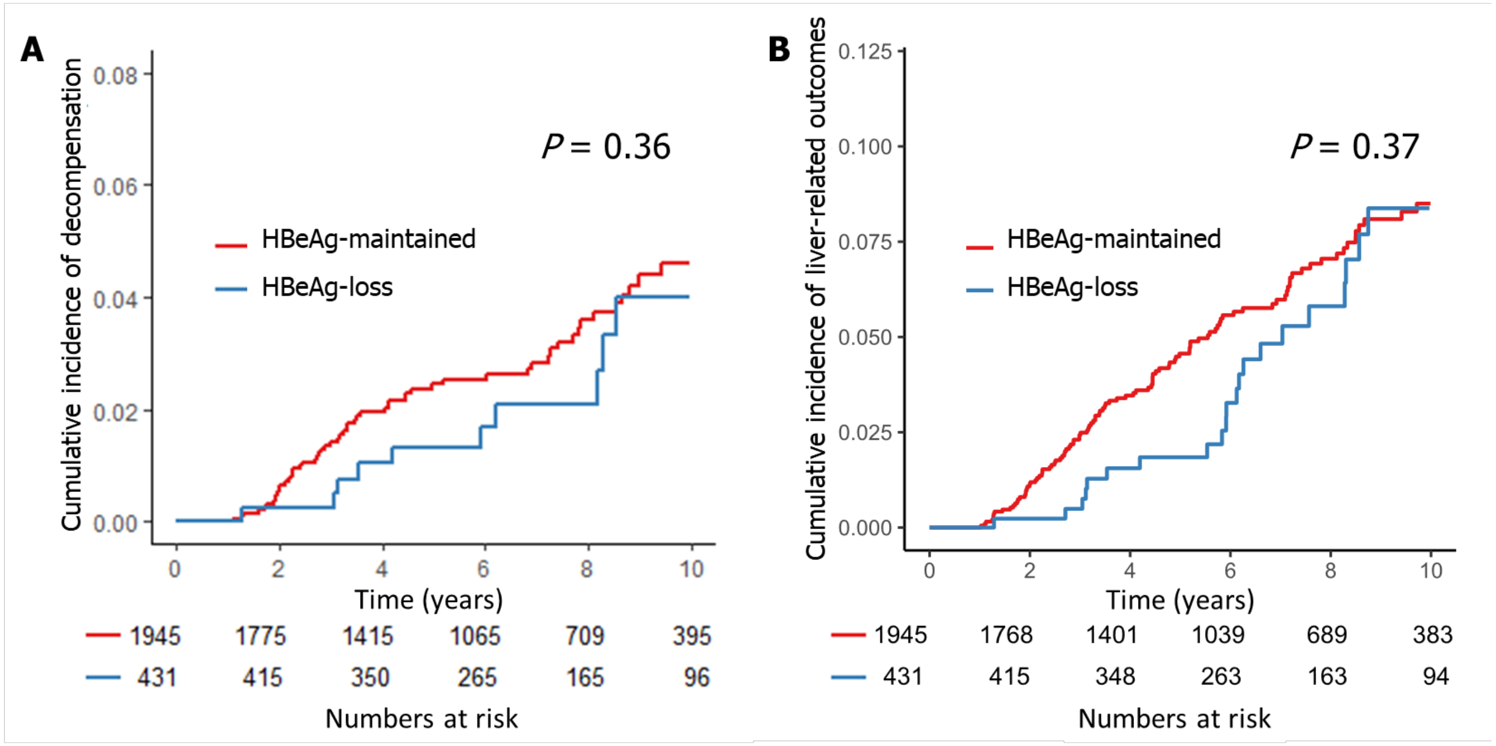

HBeAg, hepatitis B virus envelope antigen

The cumulative incidences of HCC were compared by Kaplan-Meier curves, and *P* values were derived from log-rank test.

Fig. S3. Risk of HCC according to HBeAg-seroclearance at 60 months after initiation of antiviral treatment. (A) Unweighted analysis. (B) Weight analysis balanced by inverse probability of treatment weighting.

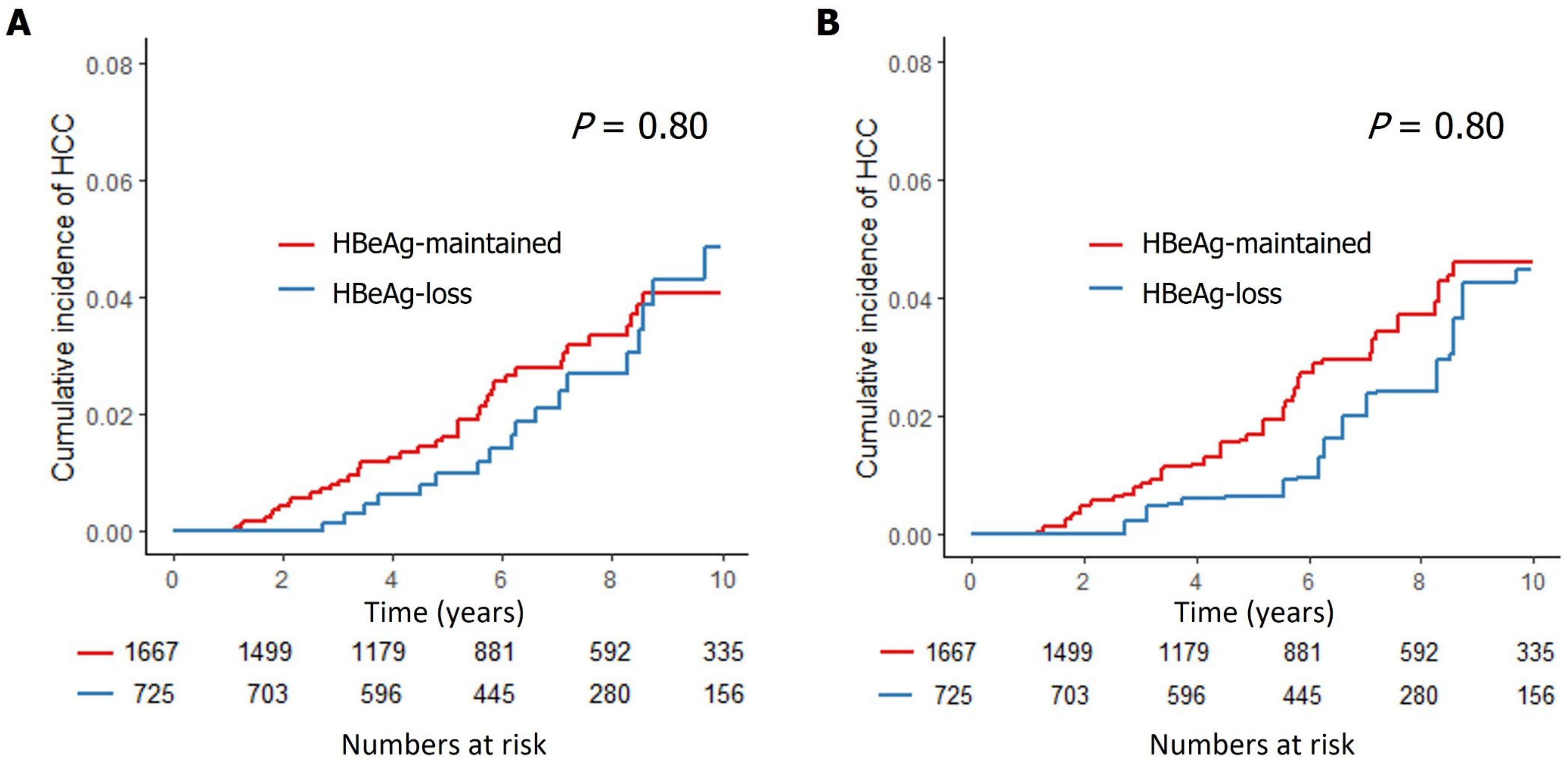

HCC, hepatocellular carcinoma; HBeAg, hepatitis B virus envelope antigen

The cumulative incidences of HCC were compared by Kaplan-Meier curves, and *P* values were derived from log-rank test.

Fig. S4. Risk of HCC according to HBeAg-seroclearance at 36 months after initiation of antiviral treatment. (A) Unweighted analysis. (B) Weight analysis balanced by inverse probability of treatment weighting in the subgroup of platelet count of more than 150×10³/µL.

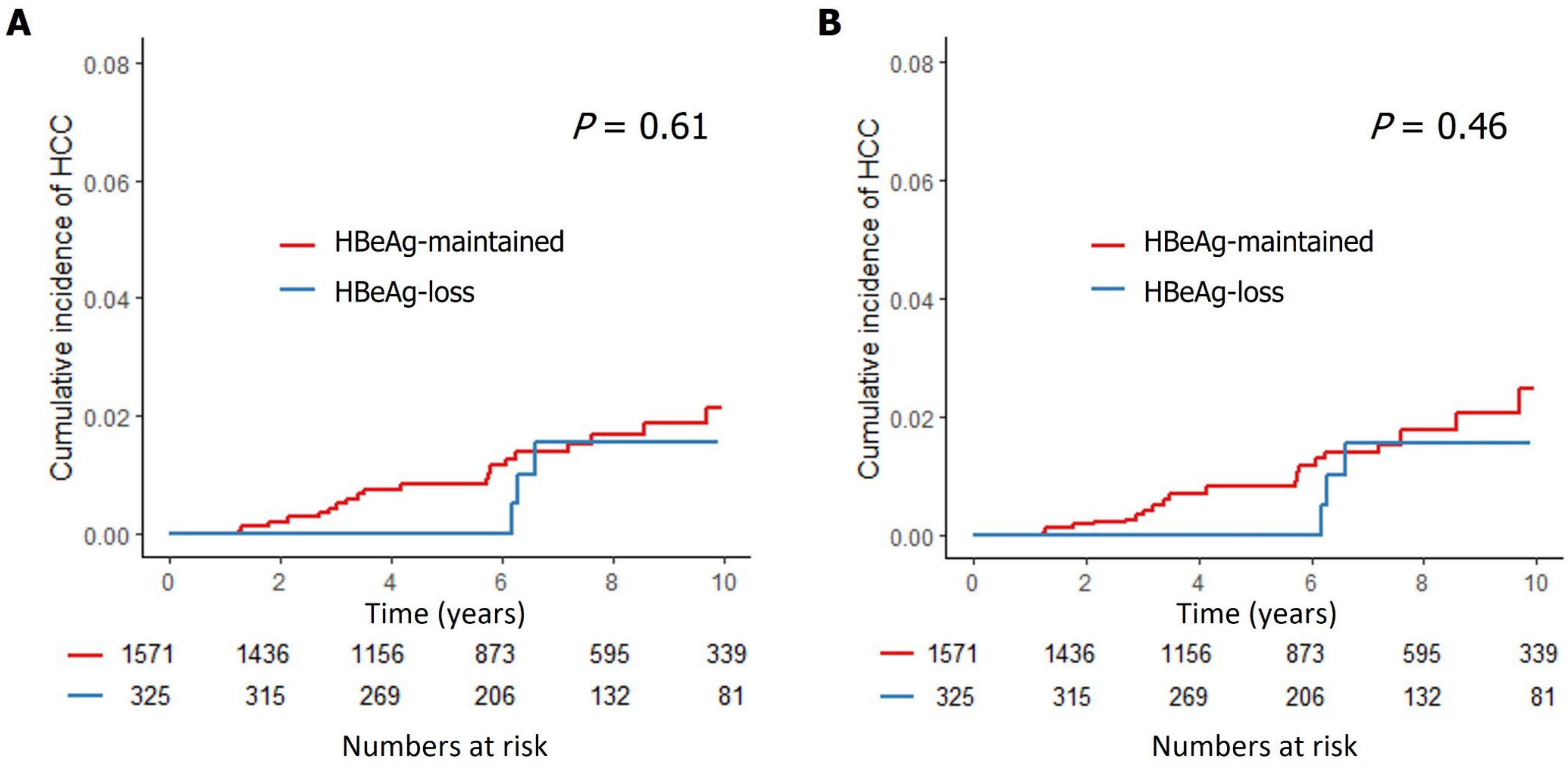

HCC, hepatocellular carcinoma; HBeAg, hepatitis B virus envelope antigen

The cumulative incidences of HCC were compared by Kaplan-Meier curves, and *P* values were derived from log-rank test.

Fig. S5. Risk of HCC according to HBeAg-seroclearance at 36 months after initiation of antiviral treatment in FIB-4 stratified groups. (A) high FIB-4 group (i.e., ≥ 2.0), and (B) low FIB-4 group (i.e., < 2.0).

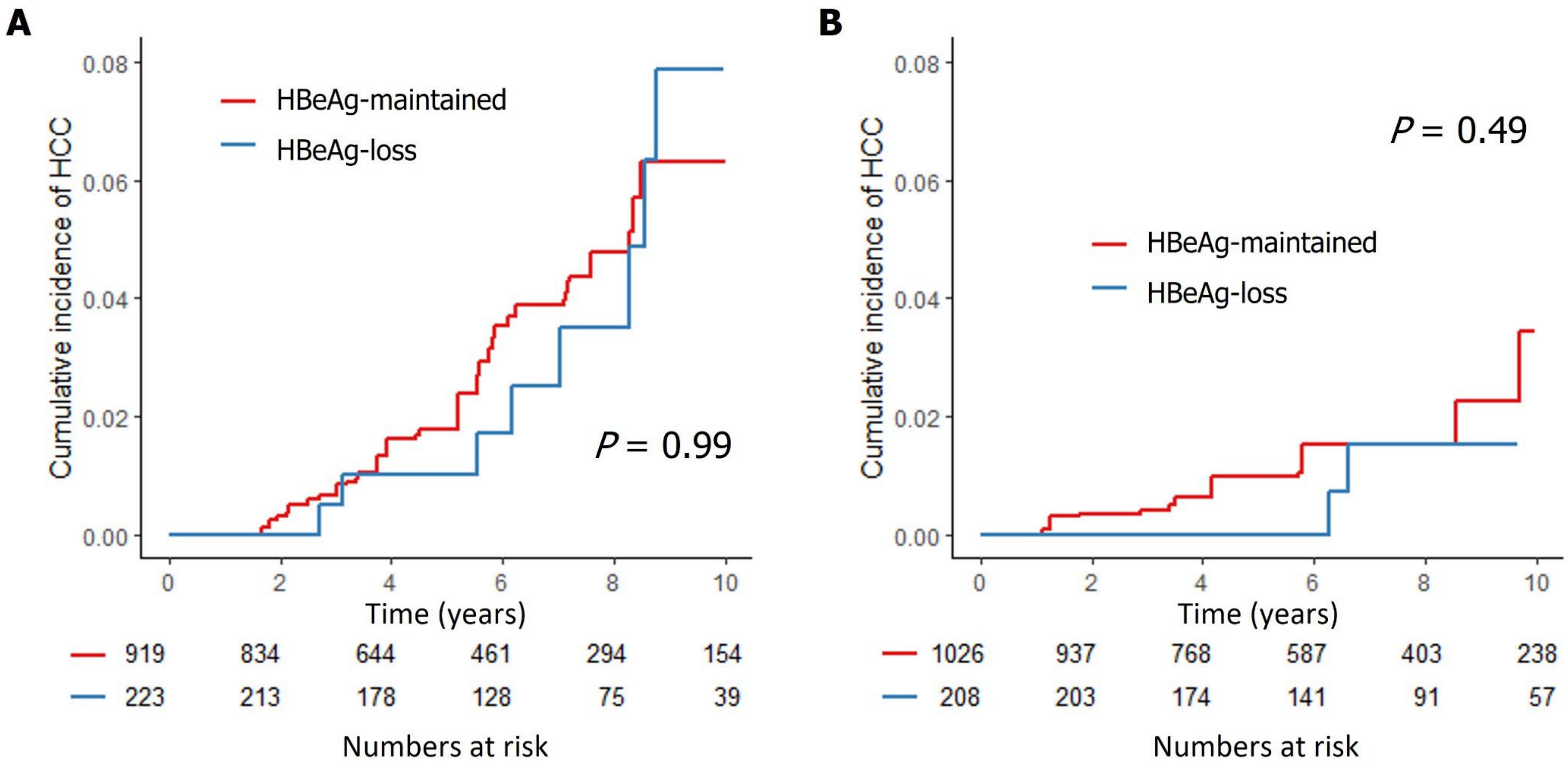

HCC, hepatocellular carcinoma; HBeAg, hepatitis B virus envelope antigen; FIB-4, fibrosis-4

The cumulative incidences of HCC were compared by Kaplan-Meier curves, and *P* values were derived from log-rank test.

Fig. S6. Risk of HCC according to HBeAg-seroclearance at 36 months after initiation of antiviral treatment in age stratified groups. (A) older age group (i.e., ≥ 45), and (B) younger age group (i.e., < 45).

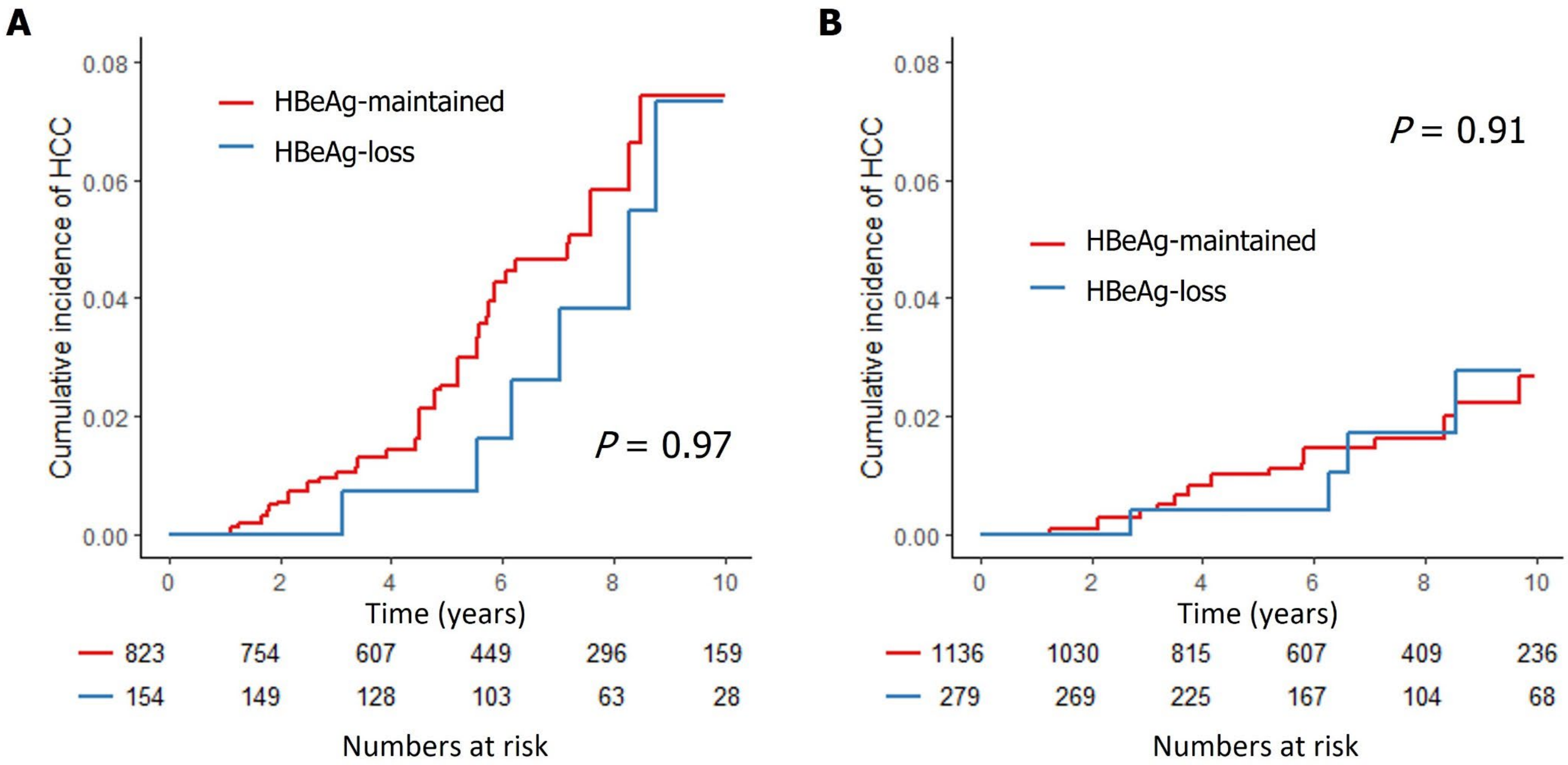

HCC, hepatocellular carcinoma; HBeAg, hepatitis B virus envelope antigen

The cumulative incidences of HCC were compared by Kaplan-Meier curves, and *P* values were derived from log-rank test.

Fig. S7. Risk of HCC according to HBV DNA suppression (i.e., serum HBV DNA ≤ 2,000 U/mL) at 4 months and baseline HBV DNA level after initiation of antiviral treatment.

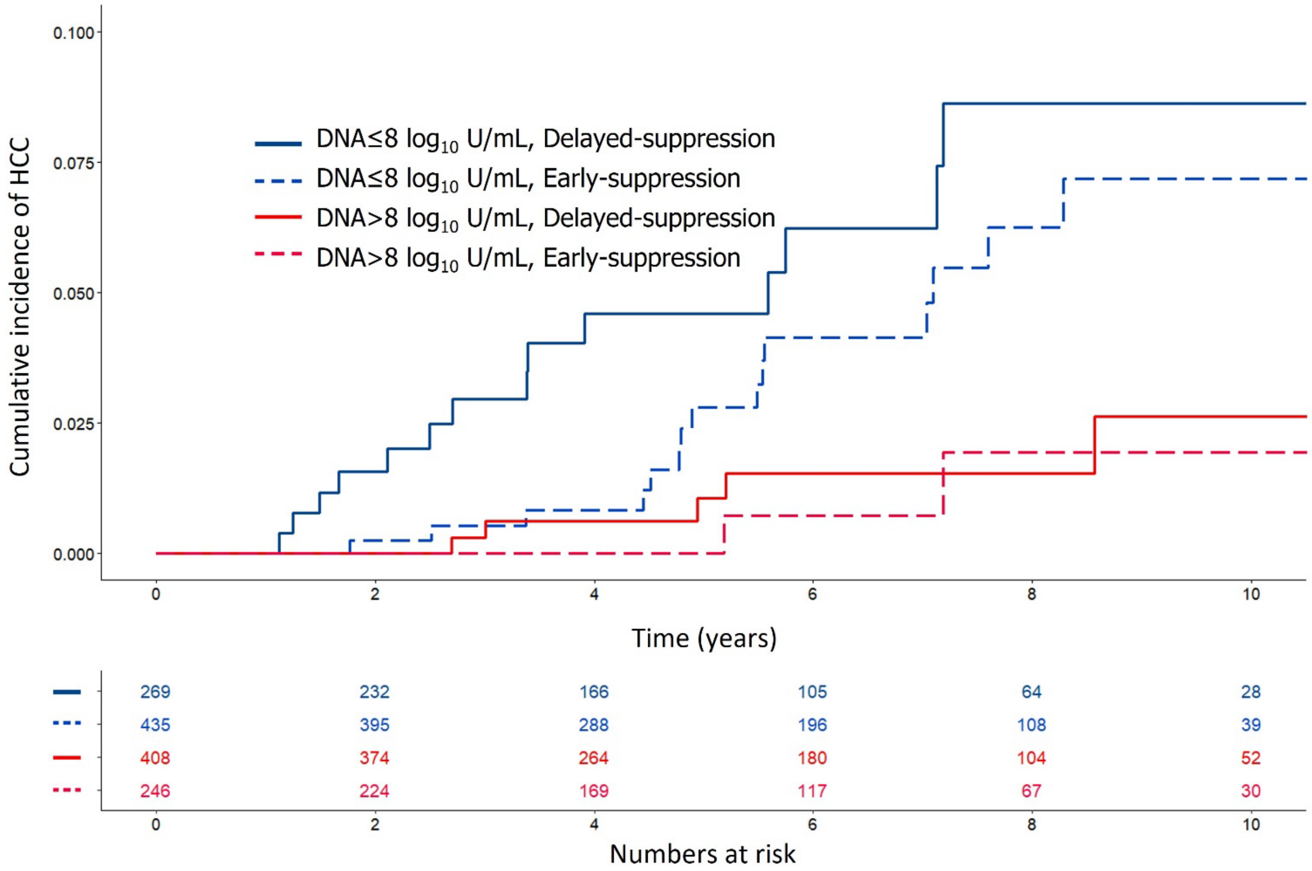

HCC, hepatocellular carcinoma

The cumulative incidences of HCC were compared by Kaplan-Meier curves, and *P* values were derived from log-rank test.

Supplementary Tables

Table S1. Baseline characteristics of the subgroup of platelet count of more than 150×10^3/µL, and the HBeAg-maintained and HBeAg-loss groups before and after inverse probability of treatment weighting.

Characteristics	Platelet > 150K	Before IPTW HBeAg-seroclearance within 36 months				After IPTW HBeAg-seroclearance within 36 months			
		HBeAg-maintained	HBeAg-loss	*P*	SMD	HBeAg-maintained	HBeAg-loss	*P*	SMD
	n = 1,896	*n* = 1,571	*n* = 325			*n* = 1,571	*n* = 325		
Age, years	40 (32–48)	40 (32–49)	37 (31–46)	<0.001	0.205	38 (31–47)	39 (32–48)	0.23	0.073
Sex, male, %	1,202 (63.4%)	1,010 (64.3%)	192 (59.1%)	0.08	0.107	998 (63.5%)	192 (59.1%)	0.14	0.090
Antiviral, %				0.50	0.043			0.53	0.039
Entecavir	1,112 (59.2%)	924 (58.8%)	198 (60.9%)			928 (59.1%)	198 (60.9%)		
TDF	774 (40.8%)	647 (41.2%)	127 (39.1%)			643 (40.9%)	127 (39.1%)		
Platelet count, 10^3/µL	203 (177–238)	204 (177–239)	200 (175–232)	0.19	0.053	202 (176–235)	200 (174–232)	0.68	0.007
Albumin, g/dL	4.1 (3.8–4.3)	4.1 (3.8–4.3)	4.1 (3.8–4.3)	0.09	0.105	4.1 (3.8–4.3)	4.1 (3.8–4.3)	0.47	0.037
AST, U/L	98 (63–179)	96 (60–167)	111 (75–234)	<0.001	0.131	111 (72–211)	111 (75–234)	0.31	0.005
ALT, U/L	130 (84–247)	125 (84–236)	157 (87–305)	<0.001	0.201	149 (90–294)	157 (86–305)	0.61	0.035
HBV DNA, $\log_{10}$ U/mL	8.0 (7.0–8.2)	8.0 (7.1–8.3)	7.9 (6.9–8.2)	<0.001	0.111	8.0 (6.9–8.2)	7.9 (6.9–8.2)	0.64	0.010
Total bilirubin, mg/dL	0.9 (0.7–1.2)	0.9 (0.7–1.2)	0.9 (0.7–1.2)	0.37	0.099	0.9 (0.7–1.2)	0.9 (0.7–1.2)	0.92	0.033
Creatinine, mg/dL	0.9 (0.7–1.0)	0.9 (0.7–1.0)	0.9 (0.8–1.0)	0.57	0.021	0.9 (0.8–1.0)	0.9 (0.8–1.0)	0.89	0.013
FIB-4	1.7 (1.1–2.6)	1.7 (1.1–2.5)	1.7 (1.1–2.8)	0.16	0.095	1.8 (1.1–2.8)	1.7 (1.1–2.8)	0.93	0.015
mPAGE-B	8 (6–10)	8 (6–10)	8 (5–10)	0.047	0.120	8 (5–10)	8 (5–10)	0.52	0.041

IPTW, inverse probability of treatment weighting; HBeAg, hepatitis B virus envelope antigen; SMD, standardized mean difference; TDF, tenofovir disoproxil fumarate; AST, aspartate aminotransferase; ALT, alanine aminotransferase; FIB-4, Fibrosis-4; mPAGE-B, modified PAGE-B

All variables were compared between the HBeAg-loss and HBeAg-maintained groups before and after IPTW. Sex, and the type of antiviral treatment were analyzed by Fisher's exact test. Other variables were analyzed by Kruskal–Wallis test and expressed in median (IQR).

Table S2. Baseline characteristics of the stratified subgroup of the FIB-4 index ≥ 2.0, and the HBeAg-maintained and HBeAg-loss groups before and after inverse probability of treatment weighting.

Characteristics		FIB-4 ≥ 2.0	Before IPTW HBeAg-seroclearance within 36 months				After IPTW HBeAg-seroclearance within 36 months			
			HBeAg-maintained	HBeAg-loss	*P*	SMD	HBeAg-maintained	HBeAg-loss	*P*	SMD
		n = 1,142	*n* = 919	*n* = 223			*n* = 919	*n* = 223		
Age, years		47 (38–55)	48 (39–55)	45 (36–53)	0.02	0.165	46 (37–54)	45 (36–53)	0.70	0.023
Sex, male, %		654 (57.3%)	537 (58.4%)	117 (52.5%)	0.11	0.120	510 (55.5%)	117 (52.5%)	0.43	0.060
Antiviral, %										
	Entecavir	673 (58.9%)	540 (58.8%)	133 (59.6%)	0.82	0.018	541 (58.9%)	133 (59.6%)	0.85	0.014
	TDF	469 (41.1%)	379 (41.2%)	90 (40.4%)			378 (41.1%)	90 (40.4%)		
Platelet count, 10^3/μL		166 (134–198)	167 (135–199)	162 (131–193)	0.18	0.084	164 (134–195)	161 (131–192)	0.63	0.023
Albumin, g/dL		3.9 (3.6–4.2)	3.9 (3.6–4.2)	3.9 (3.6–4.2)	0.26	0.064	3.9 (3.6–4.2)	3.9 (3.6–4.2)	0.32	0.063
AST, U/L		150 (90–273)	142 (88–264)	170 (96–295)	0.03	0.061	170 (97–300)	170 (95–290)	0.88	0.031
ALT, U/L		139 (82–292)	134 (82–287)	149 (84–323)	0.06	0.107	154 (88–333)	148 (84–322)	0.96	0.001
HBV DNA, $\log_{10}$ U/mL		8.0 (6.9–8.2)	8.0 (6.9–8.2)	7.8 (6.9–8.2)	0.19	0.013	8.0 (7.0–8.2)	7.8 (6.9–8.2)	0.14	0.049
Total bilirubin, mg/dL		1.0 (0.8–1.3)	1.0 (0.8–1.3)	1.0 (0.8–1.4)	0.20	0.041	1.0 (0.8–1.4)	1.0 (0.8–1.4)	0.57	0.028
Creatinine, mg/dL		0.8 (0.7–1.0)	0.9 (0.7–1.0)	0.8 (0.7–1.0)	0.81	0.105	0.8 (0.7 -1.0)	0.8 (0.7 -1.0)	1.00	0.090
FIB-4		3.4 (2.5–4.7)	3.3 (2.5–4.7)	3.6 (2.6–4.9)	0.21	0.079	3.5 (2.6–4.8)	3.6 (2.6–4.9)	0.83	0.039
mPAGE-B		10 (8–12)	10 (8–12)	10 (8–12)	0.17	0.099	10 (8–12)	10 (8–12)	0.93	0.007

IPTW, inverse probability of treatment weighting; HBeAg, hepatitis B virus envelope antigen; SMD, standardized mean difference; TDF, tenofovir disoproxil fumarate; AST, aspartate aminotransferase; ALT, alanine aminotransferase; FIB-4, Fibrosis-4; mPAGE-B, modified PAGE-B

All variables were compared between the HBeAg-loss and HBeAg-maintained groups before and after IPTW. Sex, and the type of antiviral treatment were analyzed by Fisher's exact test. Other variables were analyzed by Kruskal–Wallis test and expressed in median (IQR).

Table S3. Baseline characteristics of the stratified subgroup of the FIB-4 index < 2.0, and the HBeAg-maintained and HBeAg-loss groups before and after inverse probability of treatment weighting.

Characteristics	FIB-4 < 2.0	Before IPTW HBeAg-seroclearance within 36 months				After IPTW HBeAg-seroclearance within 36 months			
		HBeAg-maintained	HBeAg-loss	*P*	SMD	HBeAg-maintained	HBeAg-loss	*P*	SMD
	n = 1,234	*n* = 1,026	*n* = 208			*n* = 1,026	*n* = 208		
Age, years	36 (30–44)	37 (30–45)	33 (29–41)	<0.001	0.307	34 (29–42)	33 (29–41)	0.41	0.078
Sex, male, %	829 (67.2%)	690 (67.3%)	138 (66.3%)	0.81	0.021	682 (66.5%)	138 (66.2%)	0.93	0.007
Antiviral, %				0.35	0.076			0.60	0.041
Entecavir	727 (58.9%)	598 (58.3%)	129 (62%)			614 (59.8%)	129 (61.8%)		
TDF	507 (41.1%)	428 (41.7%)	79 (38%)			412 (40.2%)	79 (38.2%)		
Platelet count, $10^3/\mu L$	211 (182- 246)	212 (183–246)	203 (177–243)	0.16	0.069	206 (176–243)	203(177–242)	0.84	0.011
Albumin, g/dL	4.1 (3.9–4.4)	4.1 (3.9–4.4)	4.1 (3.9–4.3)	0.20	0.107	4.1 (3.9–4.4)	4.1 (3.9–4.3)	0.32	0.071
AST, U/L	73 (50–108)	70 (49–104)	82 (60–118)	<0.001	0.237	78 (52–127)	81 (59–118)	0.27	0.044
ALT, U/L	117 (83–198)	115 (83–191)	137 (83–247)	0.02	0.219	130 (85–237)	136 (83–247)	0.86	0.041
HBV DNA, log_{10} U/mL	8.0 (6.8–8.3)	8.0 (6.9–8.4)	7.9 (6.6–8.2)	<0.001	0.175	7.9 (6.7–8.2)	7.9 (6.6–8.2)	0.49	0.047
Total bilirubin, mg/dL	0.9 (0.7–1.1)	0.9 (0.7–1.1)	0.9 (0.7–1.1)	0.35	0.161	0.9 (0.7–1.1)	0.9 (0.7–1.1)	0.59	0.145
Creatinine, mg/dL	0.9 (0.8–1.0)	0.9 (0.8–1.0)	0.9 (0.8–1.0)	0.79	0.090	0.9 (0.8–1.0)	0.9 (0.8–1.0)	0.83	0.105
FIB-4	1.2 (0.9–1.6)	1.2 (0.9–1.6)	1.2 (0.9–1.6)	0.60	0.047	1.2 (0.9–1.6)	1.2 (0.9–1.6)	0.70	0.031
mPAGE-B	7 (5–9)	7 (5–9)	7 (5–9)	0.19	0.122	7 (5–9)	7 (5–9)	0.87	0.004

IPTW, inverse probability of treatment weighting; HBeAg, hepatitis B virus envelope antigen; SMD, standardized mean difference; TDF, tenofovir disoproxil fumarate; AST, aspartate aminotransferase; ALT, alanine aminotransferase; FIB-4, Fibrosis-4; mPAGE-B, modified PAGE-B

All variables were compared between the HBeAg-loss and HBeAg-maintained groups before and after IPTW. Sex, and the type of antiviral treatment were analyzed by Fisher's exact test. Other variables were analyzed by Kruskal–Wallis test and expressed in median (IQR).

Table S4. Baseline characteristics of the stratified subgroup of age ≥ 45, and the HBeAg-maintained and HBeAg-loss groups before and after inverse probability of treatment weighting.

Characteristics	Age ≥ 45	Before IPTW HBeAg-seroclearance within 36 months				After IPTW HBeAg-seroclearance within 36 months			
	n = 977	HBeAg-maintained *n* = 823	HBeAg-loss *n* = 154	*P*	SMD	HBeAg-maintained *n* = 823	HBeAg-loss *n* = 154	*P*	SMD
Age, years	52 (48–57)	52 (48–57)	51 (47–56)	0.10	0.095	52 (48–57)	51 (47–56)	0.28	0.067
Sex, male, %	514 (56.2%)	432 (52.5%)	82 (53.2%)	0.93	0.015	432 (52.5%)	82 (53.2%)	0.88	0.014
Antiviral, %				0.65	0.043			0.59	0.048
Entecavir	592 (60.6%)	438 (53.2%)	96 (62.3%)			494 (60.0%)	96 (62.3%)		
TDF	385 (39.4%)	385 (46.8%)	58 (37.7%)			329 (40.0%)	58 (37.7%)		
Platelet count, $10^3/\mu L$	173 (141–211)	176 (145–212)	161 (132–197)	0.01	0.145	164 (134–200)	160 (132–197)	0.65	0.006
Albumin, g/dL	3.9 (3.6–4.2)	3.9 (3.6–4.2)	3.9 (3.6–4.1)	0.15	0.102	3.9 (3.6–4.2)	3.9 (3.6–4.1)	0.41	0.076
AST, U/L	90 (62–144)	89 (62–136)	99 (66–166)	0.08	0.095	95 (65–160)	97 (66–165)	0.71	0.007
ALT, U/L	106 (72–180)	106 (72- 177)	103 (73–195)	0.97	0.004	107 (72–187)	102 (73–194)	0.84	0.015
HBV DNA, $\log_{10}$ U/mL	8.0 (6.6–8.2)	8.0 (6.6–8.2)	7.8 (6.4–8.2)	0.05	0.139	7.9 (6.6–8.2)	7.8 (6.3–8.2)	0.34	0.072
Total bilirubin, mg/dL	0.9 (0.7–1.2)	0.9 (0.7–1.2)	0.9 (0.7–1.2)	0.28	0.011	0.9 (0.7–1.2)	0.9 (0.7–1.2)	0.51	0.032
Creatinine, mg/dL	0.8 (0.7–1.0)	0.8 (0.7–1.0)	0.8 (0.7–0.9)	1.00	0.141	0.8 (0.7–1.0)	0.8 (0.7–0.9)	0.98	0.110
FIB-4	2.7 (1.8–4.2)	2.6 (1.8–4.2)	3.1 (2.1–4.7)	0.01	0.223	3.1 (2.0–4.6)	3.1 (2.1–4.7)	0.73	0.090
mPAGE-B	11 (10–13)	11 (10–13)	11 (10–13)	0.43	0.094	11 (10–13)	11 (10–13)	0.91	0.024

IPTW, inverse probability of treatment weighting; HBeAg, hepatitis B virus envelope antigen; SMD, standardized mean difference; TDF, tenofovir disoproxil fumarate; AST, aspartate aminotransferase; ALT, alanine aminotransferase; FIB-4, Fibrosis-4; mPAGE-B, modified PAGE-B

All variables were compared between the HBeAg-loss and HBeAg-maintained groups before and after IPTW. Sex, and the type of antiviral treatment were analyzed by Fisher’s exact test. Other variables were analyzed by Kruskal–Wallis test and expressed in median (IQR).

Table S5. Baseline characteristics of the stratified subgroup of age < 45, and the HBeAg-maintained and HBeAg-loss groups before and after inverse probability of treatment weighting.

Characteristics	Age < 45	Before IPTW HBeAg-seroclearance within 36 months				After IPTW HBeAg-seroclearance within 36 months			
		HBeAg-maintained	HBeAg-loss	*P*	SMD	HBeAg-maintained	HBeAg-loss	*P*	SMD
	n = 1,415	*n* = 1,136	*n* = 279			*n* = 1,136	*n* = 279		
Age, years	34 (29–39)	34 (29–39)	33 (29–38)	0.10	0.122	34 (29–39)	33 (29–38)	0.17	0.099
Sex, male, %	979 (69.2 %)	805 (70.9%)	174 (62.4%)	0.01	0.181	779 (68.6%)	96 (62.4%)	0.05	0.131
Antiviral, %									
Entecavir	822 (58.1%)	655 (57.7%)	167 (59.9%)	0.54	0.045	662 (58.3%)	167 (59.9%)	0.64	0.031
TDF	593 (41.9%)	481 (42.3%)	112 (40.1%)			474 (41.7%)	112 (40.1%)		
Platelet count, $10^3/\mu L$	200 (168–237)	202 (169–240)	194 (166–230)	0.04	0.138	198 (166–233)	194 (166–230)	0.40	0.097
Albumin, g/dL	4.1 (3.9–4.4)	4.1 (3.9–4.4)	4.1 (3.8–4.3)	0.02	0.160	4.1 (3.8–4.3)	4.1 (3.8–4.3)	0.41	0.031
AST, U/L	105 (64–206)	101 (60–195)	118 (76–246)	<0.001	0.092	120 (73–228)	118 (76–246)	0.39	<0.001
ALT, U/L	144 (88–288)	138 (87–275)	176 (96–362)	<0.001	0.182	163 (94–339)	176 (95–361)	0.35	0.048
HBV DNA, $\log_{10}$ U/mL	8.0 (7.0–8.2)	8.0 (7.1–8.2)	7.9 (7.0–8.2)	0.001	0.086	7.9 (6.9–8.2)	7.9 (6.9–8.2)	0.42	0.015
Total bilirubin, mg/dL	0.9 (0.7–1.2)	0.9 (0.7–1.2)	1.0 (0.7–1.3)	0.17	0.131	0.9 (0.7–1.3)	0.9 (0.7–1.3)	0.85	0.043
Creatinine, mg/dL	0.9 (0.8–1.0)	0.9 (0.8–1.0)	0.9 (0.8–1.0)	0.87	0.044	0.9 (0.7–1.0)	0.9 (0.8–1.0)	0.62	0.022
FIB-4	1.5 (0.9–2.5)	1.5 (0.9–2.4)	1.6 (1.1–2.7)	0.01	0.066	1.6 (1.1–2.7)	1.6 (1.1–2.7)	0.85	0.023
mPAGE-B	7 (5–9)	7 (5–9)	7 (5–9)	0.99	0.003	7 (5–9)	7 (5–9)	0.66	0.018

IPTW, inverse probability of treatment weighting; HBeAg, hepatitis B virus envelope antigen; SMD, standardized mean difference; TDF, tenofovir disoproxil fumarate; AST, aspartate aminotransferase; ALT, alanine aminotransferase; FIB-4, Fibrosis-4; mPAGE-B, modified PAGE-B

All variables were compared between the HBeAg-loss and HBeAg-maintained groups before and after IPTW. Sex, and the type of antiviral treatment were analyzed by Fisher's exact test. Other variables were analyzed by Kruskal–Wallis test and expressed in median (IQR).

Table S6. Univariable and multivariable Cox regression analyses with stratification by the timing of HBeAg-seroclearance: 1–3, 3–5, 5–7, 7–10, ≥10 years.

Characteristics	Univariable analysis			Multivariable analysis		
	**HR**[1]	**95% CI**[1]	***P***	**HR**[1]	**95% CI**[1]	***P***
Time to HBeAg-seroclearance, years						
≥ 10	1	[Reference]				
1–3	1.22	0.56, 2.68	0.62	1.05	0.46, 2.39	0.97
3–5	1.83	0.86, 3.93	0.12	1.64	0.72, 3.74	0.24
5–7	0.72	0.28, 1.86	0.49	0.74	0.28, 2.01	0.56
7–10	1.16	0.53, 2.56	0.71	1.28	0.56, 2.93	0.55
Sex, male	2.18	1.18, 4.01	0.012	3.14	1.66, 5.92	<0.001
Age, years	1.06	1.04, 1.09	<0.001	1.04	1.01, 1.07	<0.001
Platelet count, 10^3/μL						
> 250	1	[Reference]				
150–250	5.52	0.75, 40.7	0.94	4.43	0.60, 32.8	0.15
< 150	29.1	3.99, 212	<0.001	16.4	2.19, 123	0.01
ALT						
≥ 2 x ULN[1]	1	[Reference]				
1–2 x ULN[1]	3.5	2.13, 5.76	<0.001	2.71	1.56, 4.70	<0.001
HBV DNA, $\log_{10}$ U/mL	0.74	0.64, 0.85	<0.001	0.85	0.72, 0.99	0.04
FIB-4	1.14	1.08, 1.20	<0.001	1.05	0.97, 1.14	0.20

[1] 40 U/L

HCC, hepatocellular carcinoma; HR, hazard ratio; CI, confidence interval; aHR, adjusted hazard ratio; HBeAg, hepatitis B virus envelope antigen; ALT, alanine aminotransferase; ULN, upper limit of normal; FIB-4, Fibrosis-4

Multivariable Cox regression analysis was performed on covariates associated with HCC occurrence identified in univariable analysis. The results were expressed in terms of HR, 95% CI, and *P* values. This analysis included a time-dependent covariate, HBeAg seroclearance, which was the variable of interest.

Table S7. Univariable and multivariable time-dependent Cox regression analyses for the predictive factors of decompensated liver cirrhosis occurrence

Characteristics	**Univariable analysis**			**Multivariable analysis**		
	**HR**	**95% CI**	***P***	**aHR**	**95% CI**	***P***
On-treatment variables						
HBeAg-seroclearance	0.83	0.61, 1.91	0.61	0.87	0.68, 1.75	0.54
Baseline variables						
Sex, male	1.78	1.04, 3.75	0.02	2.49	1.26, 4.80	0.001
Age, years	1.07	1.03, 1.11	<0.001	1.05	1.03, 1.07	<0.001
Platelet count, $10^3/\mu L$						
>250	1	[Reference]				
150–250	4.59	0.78, 34.5	0.18	3.54	0.47, 28.1	0.30
<150	41.7	5.60, 301	<0.001	22.5	4.12, 194	0.001
ALT						
≥2 x ULN[1]	1	[Reference]				
1–2 x ULN	2.50	1.82, 5.47	<0.001	2.36	1.60, 4.98	<0.001
HBV DNA, $\log_{10}$ U/mL	0.69	0.54, 0.81	<0.001	0.78	0.68, 0.92	0.001
FIB-4	1.04	1.01, 1.09	0.003	1.02	0.96, 1.07	0.15

[1] The upper limit of normal ALT was defined as 40 IU/L.

aHR, adjusted hazard ratio; ALT, alanine aminotransferase; CI, confidence interval; FIB-4, fibrosis-4 ; HBeAg, hepatitis B virus envelope antigen; HBV, hepatitis B virus; HR, hazard ratio

Multivariable Cox regression analysis was performed on covariates associated with HCC occurrence identified in univariable analysis. The results were expressed in terms of HR, 95% CI, and *P* values. This analysis included a time-dependent covariate, HBeAg seroclearance, which was the variable of interest.

Table S8. Summarized result of HCC occurence by time-dependent Cox analyses with multiple landmarks as 24, 36, 48, 60 months after initiation of antiviral treatment.

**Cox proportional hazard analysis with IPTW-balanced groups**			
**HBeAg-seroclearance within 36 months from initiation of antivirals**			
	n	HR (95% CI)	*P*
HBeAg-loss	434	0.89 (0.47–1.68)	0.72
HBeAg-maintained	1,948		
**HBeAg-seroclearance within 60 months from initiation of antivirals**			
	n	HR (95% CI)	*P*
HBeAg-loss	725	0.85 (0.50–1.46)	0.57
HBeAg-maintained	1,657		
**Time-dependent Cox regression analysis**			
	n	HR (95% CI)	*P*
HBeAg-loss	1,077	1.10 (0.64–1.92)	0.70
HBeAg-maintained	1,305		
**Time-dependent Cox regression analysis with landmarks**			
**HBeAg-seroclearance within 24 months from initiation of antivirals**			
	n	HR (95% CI)	*P*
HBeAg-loss	261	0.57 (0.21–1.55)	0.27
HBeAg-maintained	2,121		
**HBeAg-seroclearance within 36 months from initiation of antivirals**			
	n	HR (95% CI)	*P*
HBeAg-loss	434	0.89 (0.49–1.63)	0.72
HBeAg-maintained	1,948		
**HBeAg-seroclearance within 48 months from initiation of antivirals**			
	n	HR (95% CI)	*P*
HBeAg-loss	599	1.02 (0.57–1.82)	0.95
HBeAg-maintained	1,783		
**HBeAg-seroclearance within 60 months from initiation of antivirals**			
	n	HR (95% CI)	*P*
HBeAg-loss	725	1.10 (0.63–1.93)	0.73
HBeAg-maintained	1,657		

HCC, hepatocellular carcinoma; IPTW, inverse probability of treatment weighting; HR, hazard ratio; CI, confidence interval; HBeAg, hepatitis B virus envelope antigen

Cox proportional hazard regression analysis, time-dependent Cox regression analysis, and time-dependent Cox regression analysis with landmarks were performed. The results were expressed in terms of HR, 95% CI, and *P* values.

Table S9. Baseline characteristics of the Delayed-suppression and Early-suppression groups before and after inverse probability of treatment weighting.

Characteristics		Before IPTW DNA suppression within 4 months				After IPTW DNA suppression within 4 months			
	n = 1,407	Delayed-suppression *n* = 694	Early-suppression *n* = 713	*P*	SMD	Delayed-suppression *n* = 694	Early-suppression *n* = 713	*P*	SMD
Age, years	42 (33–51)	44 (33–52)	42 (34–50)	0.480	0.032	42 (33–51)	42 (34–50)	0.98	0.007
Sex, male, %	851 (60.5%)	422 (60.8%)	429 (60.2%)	0.83	0.013	406 (58.5%)	429 (60.2%)	0.61	0.033
Antiviral, %				0.79	0.017			0.97	0.003
Entecavir	799 (56.8%)	397 (57.2%)	402 (56.4%)			390 (60.2%)	402 (56.4%)		
TDF	608 (43.2%)	297 (42.8%)	311 (43.6%)			304 (39.8%)	311 (43.6%)		
Platelet count, 10^3/μL	187 (144–227)	191 (152–230)	179 (137–226)	0.01	0.120	184 (146–227)	179 (137–226)	0.36	0.033
Albumin, g/dL	4.1 (3.8–4.4)	4.1 (3.9–4.4)	4.1 (3.8–4.3)	0.03	0.080	4.1 (3.8–4.4)	4.1 (3.8–4.3)	0.54	0.015
AST, U/L	107 (70–211)	96 (64–170)	129 (75–249)	<0.001	0.207	133 (80–255)	129 (75–249)	0.47	0.005
ALT, U/L	118 (78–248)	103 (73–193)	144 (82–293)	<0.001	0.198	143 (83–299)	144 (82–292)	0.99	0.032
HBV DNA, $\log_{10}$ U/mL	8.0 (6.7–8.2)	8.0 (7.5–8.2)	7.5 (6.1–8.2)	<0.001	0.558	7.6 (6.3–8.2)	7.5 (6.1–8.2)	0.18	0.124
Total bilirubin, mg/dL	0.9 (0.7–1.2)	0.9 (0.7–1.2)	0.9 (0.7–1.3)	0.01	0.070	0.9 (0.7–1.3)	0.9 (0.7–1.3)	0.71	0.015
Creatinine, mg/dL	0.9 (0.7–1.0)	0.9 (0.7–1.0)	0.9 (0.7–1.0)	0.63	0.007	0.9 (0.7–1.0)	0.9 (0.7–1.0)	0.63	0.023
FIB-4	2.4 (1.4–4.1)	2.3 (1.3–3.8)	2.7 (1.5–4.4)	<0.001	0.191	2.7 (1.6–4.6)	2.7 (1.5–4.3)	0.61	0.008
mPAGE-B	9 (7–11)	9 (7–11)	9 (7–12)	0.62	0.030	9 (7–11)	9 (7–12)	0.69	0.024

IPTW, inverse probability of treatment weighting; SMD, standardized mean difference; TDF, tenofovir disoproxil fumarate; AST, aspartate aminotransferase; ALT, alanine aminotransferase; FIB-4, Fibrosis-4; mPAGE-B, modified PAGE-B

All variables were compared between the HBeAg-loss and HBeAg-maintained groups before and after IPTW. Sex, and the type of antiviral treatment were analyzed by Fisher's exact test. Other variables were analyzed by Kruskal–Wallis test and expressed in median (IQR).
