# Supplementary material for: Lack of association between early on-treatment HBeAg seroclearance and development of hepatocellular carcinoma or decompensated cirrhosis
Source: JHEP Rep. 2024 Apr 8;6(7):101089. doi: 10.1016/j.jhepr.2024.101089 (PMC11225842; doi:10.1016/j.jhepr.2024.101089)
Supplement: Multimedia component 4 [file mmc4.pdf]

Lack of association between early on-treatment HBeAg seroclearance and development of hepatocellular carcinoma or decompensated cirrhosis

Hyunjae Shin[1,†], **Won-Mook Choi**[2,†], **Seung Up Kim**[3,†], Yunmi Ko[1], Youngsu Park[1], Jeayeon Park[1], Moon Haeng Hur[1], Min Kyung Park[1], Yun Bin Lee[1], Yoon Jun Kim[1], Jung-Hwan Yoon[1], Jeong-Hoon Lee[1,*], Fabien Zoulim[4]

JHEP Reports **2024**. vol. 6 | 1–8

Check for updates

Background & Aims: The association between hepatitis B envelope antigen (HBeAg) seroclearance during long-term nucleos(t) ide analogue (NA) treatment and the risk of hepatocellular carcinoma (HCC) in patients with chronic hepatitis B (CHB) remains unclear. Here, we aimed to investigate the association of HBeAg seroclearance during potent NA treatment with the development of HCC and decompensated cirrhosis.

Methods: Using a multicenter historical cohort including 2,392 non-cirrhotic adult patients with HBeAg-positive CHB who initiated NA treatment with tenofovir or entecavir, the risk of HCC and decompensated cirrhosis was compared between patients who achieved HBeAg seroclearance within 36 months of NA treatment (the HBeAg-loss group) and those who did not (the HBeAg-maintained group), using inverse probability of treatment weighting.

Results: Over a median of 6.6 years of NA treatment, 1,077 patients achieved HBeAg seroclearance (HBeAg loss rate = 6.0 per 100 person-years), 64 patients developed HCC (HCC incidence rate = 0.39 per 100 person-years), and 46 patients developed decompensated cirrhosis (decompensation incidence rate = 0.28 per 100 person-years). The HBeAg-loss and HBeAg-maintained groups had a similar risk of developing HCC (hazard ratio 0.89; 95% CI 0.47–1.68; $p = 0.72$) and decompensated cirrhosis (hazard ratio 0.98; 95% CI 0.48–1.81; $p = 0.91$). Compared with delayed HBeAg seroclearance beyond 10 years of NA treatment, the risk of HCC was comparable in those who achieved earlier HBeAg seroclearance at any time point within 10 years, regardless of baseline age and fibrotic burden.

Conclusions: Early HBeAg seroclearance during NA treatment was not associated with a reduced risk of development of HCC or decompensated cirrhosis in non-cirrhotic HBeAg-positive patients with CHB.

© 2024 The Author(s). Published by Elsevier B.V. on behalf of European Association for the Study of the Liver (EASL). This is an open access article under the CC BY license (http://creativecommons.org/licenses/by/4.0/).

Introduction

Chronic HBV infection (CHB) is the most common cause of hepatocellular carcinoma (HCC) – especially in East Asia and Africa – which is the third leading cause of cancer-related death worldwide.[1,2] By 2040, the number of deaths from HCC due to HBV is projected to double.[1,3] While antiviral treatment can reduce the risk of HCC by 45% to 63%,[4,5] it cannot eliminate the risk completely,[6,7] emphasizing the significance of identifying factors linked to on-treatment HCC risk in patients with CHB.

Seropositivity for hepatitis B envelope antigen (HBeAg), an indicator of active viral replication, has been demonstrated to be a significant risk factor for the progression of cirrhosis and HCC.[8,9] During the natural history of the disease, HBeAg seroconversion, characterized by the spontaneous loss of HBeAg and the emergence of antibodies to HBeAg, has been linked to reduced levels of HBV DNA and clinical remission of hepatitis in the majority of antiviral treatment-naïve patients with CHB.[10,11] An earlier HBeAg seroconversion is related to durable remission, reduced HBeAg reversion rates, slower progression to cirrhosis and HCC, and even increased hepatitis B surface antigen (HBsAg) seroclearance rates.[12–14]

Thus, HBeAg seroconversion has been regarded as one of the important end points in the treatment of CHB. Previous studies have demonstrated that treatment-induced HBeAg seroconversion is also associated with more favorable outcomes.[12] Patients who received treatment with interferon (IFN)-α and achieved HBeAg seroconversion showed notably longer overall survival and survival without clinical complications compared to those who maintained a positive HBeAg status.[12] A subsequent randomized-controlled trial also showed that the cumulative HCC risk was significantly lower among patients who had a higher rate of HBeAg seroconversion through IFN-α

* Corresponding author. Address: Department of Internal Medicine, Seoul National University Hospital, 101 Daehak-ro, Jongno-gu, Seoul 03080, Republic of Korea; Tel.: +82-2-2072-2228, fax: +82-2-743-6701.
E-mail addresses: pindra@empal.com, JHLeeMD@snu.ac.kr (J.-H. Lee).
† These three authors contributed equally to this study as co-first authors.
https://doi.org/10.1016/j.jhepr.2024.101089

therapy compared to the control group.[15] In contrast, a recent study involving non-cirrhotic patients who were treated with nucleos(t)ide analogues (NAs) revealed that HBeAg-positive patients with CHB had a lower incidence of HCC compared to HBeAg-negative patients, as they are in the earlier stage of the disease.[16] However, few studies have investigated whether NA treatment-induced HBeAg seroclearance correlates with HCC risk during long-term treatment with potent NAs.

Therefore, the aim of this large-scale multicenter cohort study was to comprehensively explore the association of on-treatment HBeAg seroclearance with the development of HCC and decompensated cirrhosis in non-cirrhotic, HBeAg-positive patients with CHB who were treated with high genetic barrier NAs.

Patients and methods

Study population

The source population of this study (N = 4,224) was derived from a multicenter registry of non-cirrhotic, HBeAg-positive, treatment-naïve patients with CHB who started entecavir or tenofovir disoproxil fumarate (TDF) and continued it for more than 3 months between January 2007 and December 2021 at three university-affiliated tertiary centers (Seoul National University Hospital, Asan Medical Center, and Severance Hospital) in South Korea (Fig. 1). Patients in the source population were evaluated for cirrhosis using reports of abdominal CT, MRI, or ultrasonography by certified abdominal radiologists with experience >5 years' experience. Patients were excluded if they met any of the following criteria: current or previous diagnosis of hepatitis C virus or hepatitis D virus infection, history of liver transplantation, normal alanine aminotransferase (ALT) level (*i.e.*, <40 U/L), follow-up for less than 1 year, HBeAg seroclearance or HCC occurrence within the first year after initiation of antiviral treatment, no follow-up HBeAg measurement, and age less than 20 years. Finally, a total of 2,392 patients were included in the study.

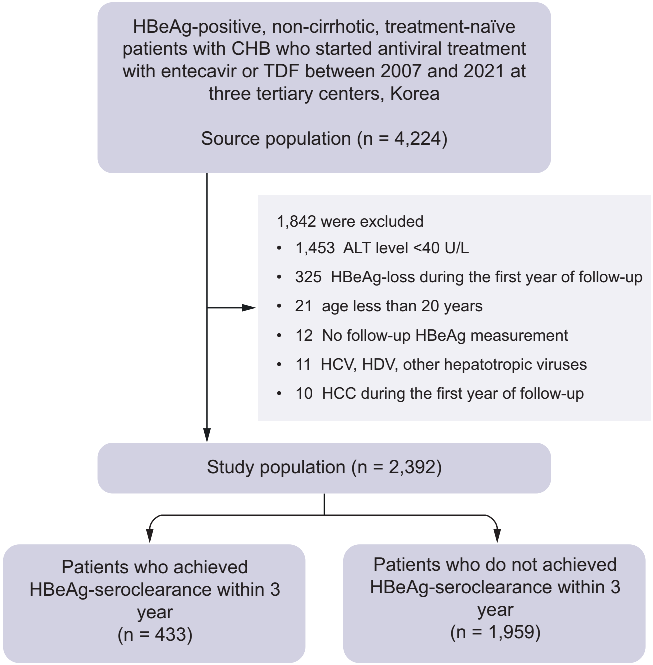

Fig. 1. Patient flow diagram. ALT, alanine aminotransferase; CHB, chronic hepatitis B; HBeAg, hepatitis B envelope antigen; HCC, hepatocellular carcinoma; TDF, tenofovir disoproxil fumarate.

The institutional review board at each participating center granted approval for this study, and due to its retrospective nature, the requirement for informed consent was waived.

Covariates and outcomes

The primary outcome was the development of HCC. The index date was defined as the date of starting ETV or TDF treatment, and the follow-up period ended at the date of HCC diagnosis, liver transplantation, death, or the date of last follow-up. All patients underwent regular HCC surveillance with ultrasonography and serum alpha-fetoprotein at the index date and every 6 months thereafter. The diagnosis of HCC was established by either histologic examination or the presence of characteristic imaging findings, such as a nodule larger than 1 cm exhibiting arterial hypervascularity and portal/delayed-phase washout, as observed on dynamic computed tomography or magnetic resonance imaging.[17,18] The secondary outcomes were the occurrence of decompensated cirrhosis and liver-related outcomes. Decompensated cirrhosis was defined as the occurrence of one or more of the following: ascites, variceal bleeding, or hepatic encephalopathy, with the prerequisite presence of cirrhosis. Liver-related outcome was defined as a composite outcome of cirrhosis, HCC, or liver-related death.

Information regarding the baseline characteristics of patients and the outcomes of the study were obtained from the electronic medical records of each participating center. The fibrosis-4 (FIB-4) index and the modified PAGE-B (mPAGE-B) score were calculated to assess the degree of fibrosis and the risk of HCC development, respectively, and for adjustment in the analysis.[19,20]

Statistical analysis

Non-parametric continuous variables are presented as medians (IQR) unless otherwise stated. Categorical variables are presented as absolute numbers of cases and/or percentages. Inverse probability of treatment weighting (IPTW) was used to balance two groups divided by the timing of HBeAg seroclearance by using the following variables: age, sex, type of antiviral drugs, ALT and aspartate aminotransferase levels, albumin, platelet counts, HBV DNA, total bilirubin, creatinine, FIB-4 index, and mPAGE-B score. Standardized mean differences (SMD) were calculated to evaluate the quality of balancing before and after IPTW. Cumulative incidence rates of HCC according to the timing of HBeAg seroclearance were evaluated using the Kaplan-Meier method and compared using the log-rank test. Univariable and multivariable Cox proportional hazard models were used to calculate hazard ratios (HRs) and 95% CIs. The timing of HBeAg seroclearance varies among patients under antiviral treatment. Time-dependent Cox regression analysis with multiple landmarks (at months 24, 36, 48, and 60) was applied to avoid immortal time bias.[21]

All data were analyzed using SPSS version 26 (IBM, Armonk, NY, USA) and R statistics version 4.2.0 (The R foundation, Vienna, Austria). Two-sided *p* values were calculated for all analyses. *p* values less than 0.05 were considered statistically significant.

Results

Baseline characteristics

A total of 2,392 HBeAg-positive patients with CHB who were treated with ETV or TDF without the occurrence of HBeAg seroclearance or HCC during the first year of the treatment were included in the analysis (Fig. 1). During a median follow-up period of 6.6 (IQR, 4.0–9.5) years, HBeAg seroclearance occurred in 1,077 patients with an annual incidence rate of 6.0% (Fig. S1). As the main analysis, patients were categorized according to whether HBeAg seroclearance occurred within 36 months of antiviral treatment (HBeAg-loss group, n = 434) or not (HBeAg-maintained group, n = 1,948). The HBeAg-loss group was younger, had lower platelet counts, albumin, and baseline HBV DNA levels, and had higher aminotransferase levels compared to the HBeAg-maintained group. After IPTW, all the baseline characteristics were well balanced, with each SMD ≤0.1 (Table 1).

IPTW analyses

During follow-up, 64 patients developed HCC (incidence rate = 0.39 per 100 person-years), 46 experienced decompensated cirrhosis (incidence rate = 0.28 per 100 person-years), and liver-related death occurred in 11 (incidence rate = 0.07 per 100 person-years). Kaplan-Meier analysis showed a similar cumulative incidence of HCC (p = 0.75, Fig. 2), decompensated cirrhosis (p = 0.36, Fig. S2A), and liver-related outcomes (p = 0.37, Fig. S2B) between the HBeAg-loss (incidence rate = 0.38 per 100 person-years) and HBeAg-maintained (incidence rate = 0.39 per 100 person-years) groups. Weighted Cox proportional hazard analysis also confirmed a comparable incidence of developing HCC (HR 0.89; 95% CI 0.47–1.68; p = 0.72), developing decompensated cirrhosis (HR 0.98; 95% CI 0.48–1.81; p = 0.91), and developing liver-related outcomes (HR 0.94; 95% CI 0.51–1.71; p = 0.95) between the two groups.

Sensitivity analyses

A sensitivity analysis was performed when the HBeAg-loss group was defined as HBeAg seroclearance within 60 months of starting antiviral treatment (n = 725), the risk of developing HCC between the two groups was similar by weighted Cox proportional hazard analysis (HR 0.85; 95% CI 0.50–1.46; p = 0.57) and by log-rank test (p = 0.80, Fig. S3).

We performed another sensitivity analysis by including only patients with a baseline platelet count ≥150,000/μl to exclude those who had a higher chance of having cirrhosis. All the baseline characteristics were well balanced after IPTW (Table S1). The results were consistent with the primary analysis by weighted Cox proportional hazard analysis (HR 0.67; 95% CI 0.23–1.91; p = 0.46; Fig. S4).

In addition, during the observation period, 61 patients experienced HBsAg loss. Among them, 37 patients had stopped NA treatment with a median follow-up period of 2.1 (IQR, 1.6–2.7) years after NA cessation. Due to the reduced risk of HCC after HBsAg seroclearance,[22] we conducted a sensitivity analysis when patients were additionally censored at the time of HBsAg loss. The weighted Cox proportional hazard analysis demonstrated a comparable risk of developing HCC between the two groups (HR 0.90; 95% CI 0.48–1.70; p = 0.81).

The IPTW analysis was conducted with the additional inclusion of patients with early HBeAg loss (less than 1 year). The risk of developing HCC between the HBeAg-loss and HBeAg-maintained groups was also comparable (HR 0.94; 95% CI 0.58–1.53; p = 0.81 by weighted Cox proportional hazard analysis; p = 0.97 by log-rank test).

Stratified analyses

First, stratified analyses were performed based on FIB-4 index (<2 and ≥2) and age (<45 and ≥45 years) to investigate the impact of HBeAg seroclearance during antiviral treatment on the risk of HCC, considering baseline fibrotic burden and age. Most of the baseline characteristics were well balanced after IPTW in all patient strata according to the FIB-4 index (Tables S2 and S3) and age (Tables S4 and S5). HBeAg seroclearance during antiviral treatment had no impact on the risk of HCC, with similar HCC risks between the HBeAg-loss group and the HBeAg-maintained group in all patient strata according to FIB-4 (p = 0.99 in the high FIB-4 group and p = 0.49 in the low FIB-4 group, Fig. S5) and age (p = 0.97 in the older age group and p = 0.91 in the younger age group, Fig. S6) by log-rank test.

Second, when the patients were stratified by the time to HBeAg seroclearance (*i.e.*, years 1–3, 3–5, 5–7, 7–10, and ≥10), the cumulative incidence of HCC did not differ significantly among groups (p = 0.20, Fig. 3). Univariable and multivariable Cox proportional hazards regression analyses were performed and showed no difference in the risk of HCC within the groups stratified by time to HBeAg seroclearance (Table S6).

Time-dependent Cox analyses

To mitigate the potential impact of immortal time bias, time-dependent Cox regression analysis, where HBeAg positivity was treated as a time-varying covariate, was performed. Patients who experienced HBeAg seroclearance during the study period (n = 1,077) had a similar risk of developing HCC (HR 1.10; 95% CI 0.64–1.92; p = 0.70; Table 2) and developing decompensated cirrhosis (HR 0.87; 95% CI 0.68–1.75; p = 0.54; Table S7) compared to patients who maintained detectable HBeAg throughout the study period (n = 1,305). Several risk factors of HCC development, such as older age, male gender, a low platelet count (<150,000/μl), and ALT levels (ranging from 1 to 2 times the upper normal limit), were noted. As shown in Table S7, in the 24-, 36-, 48-, and 60-month landmark analyses, the similar HCC risk between the HBeAg-loss and HBeAg-maintained groups was consistently observed (at month 24: HR 0.57; 95% CI 0.21–1.55; p = 0.27; at month 36: HR 0.89; 95% CI 0.49–1.63; p = 0.72; at month 48: HR 1.02; 95% CI 0.57–1.82; p = 0.95; at month 60: HR 1.10; 95% CI 0.63–1.93; p = 0.73).

Impact of HBeAg seroconversion on HCC

During follow-up, HBeAg seroconversion (*i.e.*, loss of HBeAg and development of anti-HBe) occurred in 924 (38.6%) patients at an incidence rate of 5.1%. An IPTW analysis comparing patients with HBeAg seroconversion within 36 months (n = 216, 9.0%) to those without seroconversion within 36 months (n = 2,176) showed a comparable incidence of HCC (HR 0.92; 95% CI 0.44–1.82; p = 0.79) by time-dependent Cox regression analysis. In addition, risk of HCC development was compared

Table 1. Baseline characteristics of the study population, HBeAg-maintained group and HBeAg-loss group.

		Before IPTW				After IPTW			
		HBeAg seroclearance within 36 months				HBeAg seroclearance within 36 months			
	All	HBeAg-maintained	HBeAg-loss			HBeAg-maintained	HBeAg-loss		
Characteristics	n = 2,392	n = 1,959	n = 433	*p* value	SMD	n = 1,959	n = 433	*p* value	SMD
Age, years	41 (33–55)	41 (33–51)	39 (32–48)	0.001	0.173	40 (32–49)	39 (32–48)	0.76	0.050
Sex*, male, %	1,492 (62.4%)	1,234 (63.0%)	258 (59.6%)	0.14	0.080	1,226 (62.6%)	257 (59.4%)	0.24	0.064
Antiviral*, %				0.44	0.043			0.40	0.046
Entecavir	1,414 (59.1%)	1,150 (58.7%)	263 (60.8%)			1,148 (58.6%)	263 (60.8%)		
TDF	978 (40.9%)	809 (41.3%)	170 (39.2%)			811 (41.4%)	170 (39.2%)		
Platelet count, $10^3/\mu l$	191 (157–228)	192 (158–229)	185 (150–222)	0.01	0.110	185 (152–221)	185 (150–222)	0.66	0.011
Albumin, g/dl	4.1 (3.8–4.3)	4.1 (3.8–4.3)	4.0 (3.7–4.3)	0.04	0.102	4.0 (3.7–4.0)	4.0 (3.7–4.0)	0.55	0.042
AST, U/L	98 (63–181)	95 (61–171)	111 (71–223)	<0.001	0.104	110 (71–213)	110 (71–222)	0.67	0.001
ALT, U/L	126 (83–239)	121 (82–230)	146 (84–288)	0.001	0.156	144 (87–286)	146 (84–288)	0.85	0.002
HBV DNA, $\log_{10}$ U/ml	8.0 (6.9–8.2)	8.0 (6.9–8.2)	7.8 (6.8–8.2)	<0.001	0.101	7.8 (6.7–8.0)	7.8 (6.8–8.0)	0.77	0.007
Total bilirubin, mg/dl	0.9 (0.7–1.2)	0.9 (0.7–1.2)	1.0 (0.7–1.3)	0.06	0.097	0.9 (0.7–1.0)	1.0 (0.7–1.0)	0.80	0.038
Creatinine, mg/dl	0.9 (0.7–1.0)	0.9 (0.7–1.0)	0.9 (0.7–1.0)	0.82	0.070	0.9 (0.7–1.0)	0.9 (0.7–1.0)	0.88	0.042
FIB-4	1.9 (1.2–3.3)	1.9 (1.2–3.2)	2.1 (1.2–3.6)	0.06	0.097	2.1 (1.3–3.0)	2.1 (1.2–4.0)	0.94	0.045
mPAGE-B	9 (6–11)	9 (6–11)	9 (6–11)	0.25	0.061	9 (6–11)	9 (6–11)	0.76	0.012

ALT, alanine aminotransferase; AST, aspartate aminotransferase; FIB-4, fibrosis-4; HBeAg, hepatitis B envelope antigen; IPTW, inverse probability of treatment weighting; mPAGE-B, modified PAGE-B; TDF, tenofovir disoproxil fumarate; SMD, standardized mean difference.
All variables were compared between the HBeAg-loss and HBeAg-maintained groups before and after IPTW. Sex, and the type of antiviral treatment were analyzed by Fisher's exact test. Other variables were analyzed by Kruskal–Wallis test and expressed in median (IQR).

between the patients who had HBeAg seroconversion and patients who had HBeAg seroclearance without seroconversion (n = 153), and found no statistical differences (HR 1.02; 95% CI 0.34–2.52; p = 0.89) by weighted Cox regression analysis.

Impact of DNA suppression on HCC

Another analysis was conducted to determine the effect of the timing of HBV DNA suppression (*i.e.*, serum HBV DNA ≤2,000 U/ml). Of the study cohort, the timing of the initial DNA suppression following the initiation of antiviral treatment was available in 1,407 patients and DNA suppression was achieved at a median of 3.9 (IQR, 2.8–6.0) months among the patients. Patients were categorized according to whether HBV DNA suppression occurred within 4 months of antiviral treatment (*i.e.*, early-suppression and delayed-suppression groups). After IPTW, all baseline characteristics were well balanced, with each SMD ≤0.1 (Table S8). The cumulative incidence of HCC was lower in the early-suppression group with low baseline DNA level (*i.e.*, HBV DNA ≤8 $\log_{10}$ U/ml) than the delayed-suppression group with low baseline DNA level (p = 0.04, Fig. S7).

Discussion

In this multicenter cohort study involving 2,392 treatment-naïve, non-cirrhotic, HBeAg-positive patients with CHB, we investigated the impact of HBeAg seroclearance during antiviral treatment with potent NAs on the risk of HCC. Our findings indicate that early on-treatment HBeAg seroclearance within 3 years was not associated with the development of HCC.

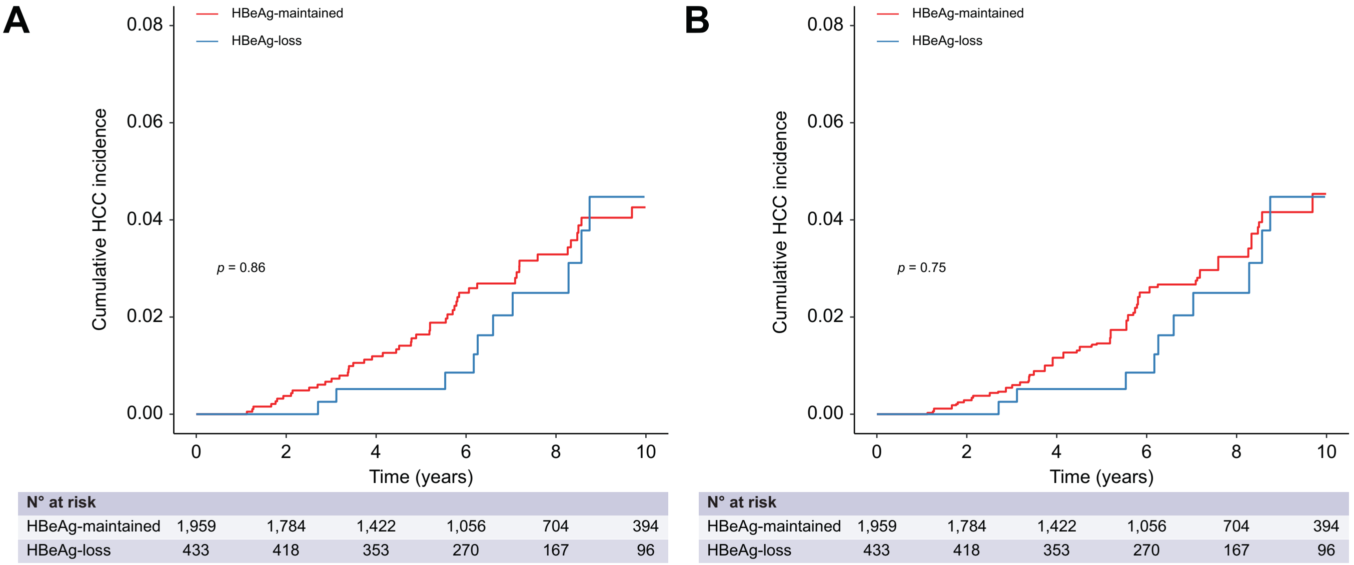

Fig. 2. Risk of HCC according to HBeAg seroclearance at 36 months after initiation of antiviral treatment. (A) Unweighted analysis. (B) Weight analysis balanced by inverse probability of treatment weighting. The cumulative incidences of HCC were compared by Kaplan-Meier curves, and p values were derived from log-rank test. HBeAg, hepatitis B envelope antigen; HCC, hepatocellular carcinoma.

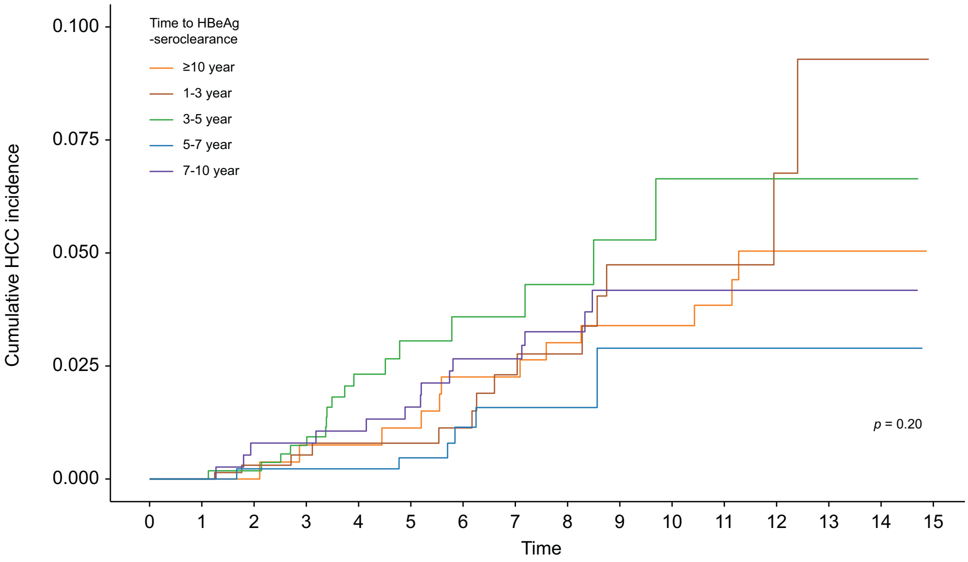

N° at risk																
≥10 year	266	266	266	264	264	263	259	258	256	253	247	176	113	66	20	0
1-3 year	753	753	578	383	353	322	270	214	167	128	96	62	46	23	12	0
3-5 year	549	549	541	521	364	209	175	139	113	83	60	39	27	18	6	0
5-7 year	446	446	443	434	421	407	261	109	89	63	47	34	19	7	4	0
7-10 year	378	378	374	374	373	370	361	350	247	139	41	26	20	15	6	0

Fig. 3. Cumulative incidence of hepatocellular carcinoma stratified by the timing of HBeAg seroclearance. The cumulative incidences of HCC were compared by Kaplan-Meier curves, and *p* values were derived from log-rank test. HCC, hepatocellular carcinoma.

Compared with delayed HBeAg seroclearance beyond 10 years of antiviral treatment, there was no difference in the risk of patients with earlier HBeAg seroclearance at years 1–3, 3–5, 5–7, and 7–10 during the overall follow-up period. These results were consistently observed in IPTW analysis and time-dependent Cox analysis. Furthermore, in sensitivity analyses where the landmark time or the cut-off for platelet count to define cirrhosis was modified, the results remained consistent. Additionally, in stratified analyses, the risk of HCC was comparable between the HBeAg-loss group and the HBeAg-maintained group, irrespective of baseline age, serum levels of ALT and HBV DNA, and fibrotic burden.

Till now, the serological response of HBeAg seroclearance, as well as virological and biochemical responses, was considered an important endpoint of NA treatment because HBeAg positivity has been identified as a risk factor for HCC in both treatment-naïve patients and those treated with IFN- α in previous studies. A long-term prospective study on Taiwanese men found a sixfold higher risk of HCC development in association with HBeAg positivity,[8] and another case-control study

Table 2. Univariable and multivariable time-dependent Cox regression analyses for the predictive factors of HCC occurrence.

	Univariable analysis			Multivariable analysis		
**Characteristics**	**HR**	**95% CI**	***p* value**	**aHR**	**95% CI**	***p* value**
**On-treatment variables**						
HBeAg seroclearance	1.17	0.69, 2.01	0.56	1.10	0.64, 1.92	0.72
**Baseline variables**						
Sex, male	2.18	1.18, 4.01	0.01	3.13	1.66, 5.92	<0.001
Age, years	1.06	1.04, 1.08	<0.001	1.04	1.02, 1.07	<0.001
Platelet count, $10^3/\mu l$						
>250	1 [Reference]					
150–250	5.07	0.69, 37.5	0.11	3.87	0.52, 28.8	0.19
<150	29.6	4.07, 216	<0.001	16.7	2.23, 125	0.01
ALT						
≥2 × ULN[1]	1 [Reference]					
1–2 x ULN	3.50	2.13, 5.76	<0.001	2.66	1.53, 4.60	<0.001
HBV DNA, log_{10} IU/ml	0.74	0.64, 0.85	<0.001	0.84	0.72, 0.99	0.03
FIB-4	1.14	1.08, 1.20	<0.001	1.05	0.97, 1.14	0.23

aHR, adjusted hazard ratio; ALT, alanine aminotransferase; FIB-4, fibrosis-4; HBeAg, hepatitis B envelope antigen; HCC, hepatocellular carcinoma; HR, hazard ratio; ULN, upper limit of normal. Multivariable Cox regression analysis was performed on covariates associated with HCC occurrence identified in univariable analysis. The results were expressed in terms of HR, 95% CI, and *p* values. This analysis included a time-dependent covariate, HBeAg seroclearance, which was the variable of interest.
[1]The upper limit of normal ALT was defined as 40 IU/L.

reported a significantly increased HCC risk in patients positive for both HBsAg and HBeAg.[23] Conversely, spontaneous or treatment-induced HBeAg seroclearance, particularly with IFN therapy, has been linked to improved long-term outcomes, including a reduced risk of cirrhosis and HCC, higher survival rates, and increased HBsAg seroclearance.[10,11,13–15] However, there has been limited data studying the impact of NA-induced HBeAg seroclearance on the risk of HCC in HBeAg-positive patients with CHB. Only a few studies, like the current one, found that there was no association between the risk of HCC and HBeAg seroclearance. In a historical cohort study that included 4,639 patients initiating ETV or TDF treatment, HBeAg seroclearance at 2 years of treatment had no impact on the risk of HCC during the overall follow-up period.[24] A recent Hong Kong study involving 1,400 NA-treated patients with CHB found that high on-treatment hepatitis B core-related antigen (HBcrAg) titers were associated with a higher risk of HCC in HBeAg-negative patients, but not in HBeAg-positive patients.[25] Given that HBeAg is the main constituent of HBcrAg in HBeAg-positive patients with CHB, it could be postulated that a decrease in HBeAg titer during antiviral treatment is not associated with a reduced risk of HCC in HBeAg-positive patients with CHB. Of note, the absence of a recent prediction model for HCC in NA-treated patients with CHB that incorporates HBeAg as a variable indirectly suggests that HBeAg positivity may not be associated with the development of HCC.[26,27]

NA is generally not regarded as having an inherent immune regulatory effect by itself.[28] Thus, there appears to be a fundamental difference between NA-induced HBeAg seroclearance *vs.* spontaneous or IFN-induced HBeAg seroclearance, which is driven by enhanced host immunity. When compared with spontaneous induction or IFN treatment, the incidence of HBeAg seroclearance was lower in patients treated with NA.[29] Additionally, the duration of HBeAg seroclearance induced by NA treatment was shorter compared to that achieved with IFN.[30] NA treatment can potentially enhance the reactivity of HBV-specific T cells. However, these drugs may also decrease the amount of viral antigens, which are necessary to stimulate immune responses. During early lamivudine treatment, HBV-specific T cells were detected, but their activity was partial, temporary, and usually disappeared within about 6 months, without a significant increase in the HBeAg seroconversion rate.[31] The use of more potent NAs did not appear to enhance the effects of increasing the rate of HBeAg seroclearance.[29] Our observation suggests that HBV DNA levels are the major driver of HCC risk. In the natural history of the infection or in IFN-treated patients, HBV DNA suppression is usually associated with HBeAg seroconversion. Those who do not clear HBeAg, usually have higher HBV DNA level on average. In contrast, HBV DNA is potently suppressed in patients treated with NAs, rendering HBeAg seroclearance irrelevant.

The exact mechanism by which NA treatment leads to a decrease in HBeAg levels remains uncertain. It is possible that the reduction in HBeAg levels results from the death of infected cells, the prevention of new rounds of HBV infection, and the replacement of infected cells with non-infected cells, which may further diminish the intrahepatic covalently closed circular DNA (cccDNA) pool. HBeAg seroconversion is not only associated with a decrease in the cccDNA pool but also with a reduction in transcriptional activity.[32] HBeAg seroclearance in patients undergoing NA treatment can also be explained by negative frequency-dependent selection. Genetic drift, which refers to random fluctuations in the relative frequencies of genetic variants (such as the wild-type virus and HBeAg-negative mutant viruses) within a population, can either enhance or counteract the impact of negative frequency-dependent selection.[33] These fluctuations occur when there is a small effective population size, defined as the number of replicating virions in an 'ideal' population that would exhibit the same degree of genetic drift as the non-ideal population under study, with amplified sampling effects. If NA effectively reduces viral replication to a level where the daily production of virions from each cccDNA is balanced, it leads to an increased effective population size and a reduction in genetic drift. As a result, the higher frequency of HBeAg-negative mutants with a replicative advantage, stemming from energy savings due to the absence of HBeAg translation or reduced transcription of precore mRNA, may lead to HBeAg seroclearance.[34,35] A previous observation that patients with HBeAg seroconversion had a higher level of full genome diversity at baseline may indirectly support this hypothesis of the negative frequency-dependent selection of HBeAg-negative mutants.[33] Given the association between HBeAg-negative mutants carrying precore/core mutations and an increased risk of HCC,[36] it is speculated that the increased HCC risk associated with these mutants counteracts the risk reduction attributed to the reduction of cccDNA achieved through NA treatment. From this perspective, it is suggested that HBeAg serves as an indicator of the early virologic phase of CHB rather than a direct carcinogen in non-cirrhotic patients undergoing NA treatment.[16] Therefore, if the objective is to prevent HCC development, achieving HBeAg seroclearance in the context of NA treatment is unlikely to be an appropriate surrogate endpoint.

Most risk factors identified from univariable and multivariable analyses are consistent with well-established risk factors reported in prior studies.[37–39] While we identified several risk factors for HCC, the impact of some, including FIB-4, has not been conclusively determined. FIB-4, similar to platelet count, could serve as an indicator of fibrotic burden. However, focusing on patients with a median FIB-4 index of 1.9 presented challenges in accurately assessing how each unit increase in FIB-4 affects the risk of developing HCC.[40] Additionally, the median HBV DNA level was measured as 8.0 $\log_{10}$ IU/ml in our cohort, which corresponds to the margin of the parabolic association between DNA and HCC risk identified in a recent study.[41] Therefore, it may have been difficult to assess the impact of DNA levels on risk through Cox regression analysis due to its parabolic association. Unlike HBV DNA, which is known to influence the progression of decompensated cirrhosis, the impact of HBeAg on disease progression is not well understood.[42,43] In our study, we were able to identify the effects of baseline fibrotic burden and HBV DNA through multivariable analysis. However, we were unable to ascertain the impact of HBeAg seroclearance as a time-dependent variable.

Our study has several limitations. First, as an observational study, our results may be susceptible to bias and confounding. Particularly, since HBeAg, anti-HBe, and HBV DNA were not regularly monitored during the study period according to pre-

established protocols, there might be an inevitable bias from the consistency assumption, which might weaken the causal claim of our study.[44] To mitigate these limitations, we applied rigorous statistical methods, including IPTW analysis, time-dependent Cox analysis, and landmark analysis, and conducted various sensitivity and stratified analyses.[45,46] Given the relatively low incidence of HCC and liver-related outcomes in non-cirrhotic patients with CHB, an observational study with a large sample size (such as ours) may serve as the only alternative to a prospective study or a randomized-controlled trial for evaluating HCC risk factors in this patent population.[47] Second, as a single nation study, this study included only Korean patients who are predominantly infected with genotype C,[48] which is associated with a higher risk of HCC,[49] through vertical transmission. The generalizability of our findings to patients with different genotypes of HBV may be limited. However, it should be noted that such a study was possible only in Korea because a significant number of patients with genotype C infection, which is associated with delayed HBeAg seroclearance,[50] initiate NA treatment during the HBeAg-positive CHB phase. Third, our study lacks data on new emerging biomarkers for HBV, such as HBV RNA, HBcrAg, and quantitative HBsAg, which were not routinely tested in clinical practice. These biomarkers could have acted as a confounder in the association between HBeAg seroclearance during antiviral treatment and the risk of HCC development. Further investigations might be warranted in patients with different HBV genotypes and ethnicities, as well as several baseline novel biomarkers.

In conclusion, our comprehensive analysis, including a large number of non-cirrhotic, HBeAg-positive patients with CHB treated with ETV or TDF, found that HBeAg seroclearance during antiviral treatment was not associated with on-treatment HCC risk, regardless of the timing of HBeAg seroclearance. Although the underlying mechanism is not fully understood, our findings indicate that targeting HBeAg seroclearance as an endpoint for NA treatment may not be appropriate in the context of HCC and decompensated cirrhosis prevention. Further studies are warranted to validate our findings.

Affiliations

[1]Department of Internal Medicine and Liver Research Institute, Seoul National University College of Medicine, Seoul, Republic of Korea; [2]Department of Gastroenterology, Liver Center, Asan Medical Center, University of Ulsan College of Medicine, Seoul, Republic of Korea; [3]Department of Internal Medicine, Yonsei University College of Medicine, Seoul, Korea; Yonsei Liver Center, Severance Hospital, Seoul, Republic of Korea; [4]INSERM Unit 1052 - Cancer Research Center of Lyon, Hospices Civils de Lyon, Lyon University, Lyon, France

Abbreviations

CHB, chronic hepatitis B; FIB-4, Fibrosis-4; HCC, hepatocellular carcinoma; HBcrAg, hepatitis B core-related antigen; HBeAg, hepatitis B envelope antigen; HBsAg, hepatitis B surface antigen; IFN, interferon; IPTW, inverse probability of treatment weighting; mPAGE-B, modified PAGE-B; NA, nucleos(t)ide analogue; TDF, tenofovir disoproxil fumarate.

Financial support

This work was supported by the National IT Industry Promotion Agency, Republic of Korea, under project name Doctor Answer 2.0 and project code S0252-21-1001.

Conflict of interest

Dr. Yun Bin Lee reports receiving research grants from Samjin Pharmaceuticals and Yuhan Pharmaceuticals; Dr. Yoon Jun Kim reports receiving research grants from Bristol-Myers Squibb, Roche, JW Creagene, Bukwang Pharmaceuticals, Handok Pharmaceuticals, Hanmi Pharmaceuticals, Yuhan Pharmaceuticals, and Pharmaking, and lecture fees from Bayer HealthCare Pharmaceuticals, Gilead Science, MSD Korea, Yuhan Pharmaceuticals, Samil Pharmaceuticals, CJ Pharmaceuticals, Bukwang Pharmaceuticals, and Handok Pharmaceuticals; Dr. Jung-Hwan Yoon reports receiving research grants from Bayer HealthCare Pharmaceuticals, Daewoong Pharmaceuticals, and Bukwang Pharmaceuticals; Dr. Jeong-Hoon Lee reports receiving research grants from Yuhan Pharmaceuticals, and lecture fee from GreenCross Cell, Daewoong Pharmaceuticals, and Gilead Korea; All other authors have declared that no conflict of interest exists.

Please refer to the accompanying ICMJE disclosure forms for further details.

Authors' contributions

J.-H.L. had full access to all of the data of this study and take responsibility for the integrity of the data and the accuracy of the data analysis. H.S., W.-M.C., S.U.K. collected the data and performed the statistical analysis. Y.K., Y.P., J.P., M.H.H., M.K.P. collected the data and performed the data analysis and interpretation. Y.B.L., Y.J.K., J.-H.Y. and J.-H.L. reviewed the data. J.-H.L. and F.Z. conceptualized and supervised the manuscript. H.S., W.-M.C., S.U.K. and J.-H.L. wrote the manuscript with comments from all authors.

Data availability statement

Data are available upon reasonable request. Data generated or analysed during the study are available from the corresponding author by request.

Supplementary data

Supplementary data to this article can be found online at https://doi.org/10.1016/j.jhepr.2024.101089.

References

Author names in bold designate shared co-first authorship

[1] Dusheiko G, Agarwal K, Maini MK. New approaches to chronic hepatitis B. N Engl J Med 2023;388:55–69.
[2] Cui F, Blach S, Manzengo Mingiedi C, et al. Global reporting of progress towards elimination of hepatitis B and hepatitis C. Lancet Gastroenterol Hepatol 2023;8:332–342.
[3] Thomas DL. Global elimination of chronic hepatitis. N Engl J Med 2019;380:2041–2050.
[4] Wu CY, Lin JT, Ho HJ, et al. Association of nucleos(t)ide analogue therapy with reduced risk of hepatocellular carcinoma in patients with chronic hepatitis B-A nationwide cohort study. Gastroenterology 2014;147:143–151.
[5] Wong GL, Chan HL, Mak CW, et al. Entecavir treatment reduces hepatic events and deaths in chronic hepatitis B patients with cirrhosis. Hepatology 2013;58:1537–1547.
[6] Papatheodoridis GV, Chan HL, Hansen BE, et al. Risk of hepatocellular carcinoma in chronic hepatitis B: assessment and modification with current antiviral therapy. J Hepatol 2015;62:956–967.
[7] Arends P, Sonneveld MJ, Zoutendijk R, et al. Entecavir treatment does not eliminate the risk of hepatocellular carcinoma in chronic hepatitis B: limited role for risk scores in Caucasians. Gut 2015;64:1289–1295.
[8] Yang HI, Lu SN, Liaw YF, et al. Hepatitis B e antigen and the risk of hepatocellular carcinoma. N Engl J Med 2002;347:168–174.
[9] Chen CJ, Yang HI, Su J, et al. Risk of hepatocellular carcinoma across a biological gradient of serum hepatitis B virus DNA level. JAMA 2006;295:65–73.
[10] Hsu YS, Chien RN, Yeh CT, et al. Long-term outcome after spontaneous HBeAg seroconversion in patients with chronic hepatitis B. Hepatology 2002;35:1522–1527.
[11] Chu CM, Hung SJ, Lin J, et al. Natural history of hepatitis B e antigen to antibody seroconversion in patients with normal serum aminotransferase levels. Am J Med 2004;116:829–834.
[12] Niederau C, Heintges T, Lange S, et al. Long-term follow-up of HBeAg-positive patients treated with interferon alfa for chronic hepatitis B. N Engl J Med 1996;334:1422–1427.

[13] Lin SM, Yu ML, Lee CM, et al. Interferon therapy in HBeAg positive chronic hepatitis reduces progression to cirrhosis and hepatocellular carcinoma. J Hepatol 2007;46:45–52.
[14] Chen YC, Chu CM, Liaw YF. Age-specific prognosis following spontaneous hepatitis B e antigen seroconversion in chronic hepatitis B. Hepatology 2010;51:435–444.
[15] Lin SM, Sheen IS, Chien RN, et al. Long-term beneficial effect of interferon therapy in patients with chronic hepatitis B virus infection. Hepatology 1999;29:971–975.
[16] **Jang H**, **Yoon JS**, **Park SY**, et al. Impact of HBeAg on hepatocellular carcinoma risk during oral antiviral treatment in patients with chronic hepatitis B. Clin Gastroenterol Hepatol 2022;20:1343–1353.e1316.
[17] European Association for the Study of the Liver. EASL clinical practice guidelines: management of hepatocellular carcinoma. J Hepatol 2018;69: 182–236.
[18] Marrero JA, Kulik LM, Sirlin CB, et al. Diagnosis, staging, and management of hepatocellular carcinoma: 2018 practice guidance by the American association for the study of liver diseases. Hepatology 2018;68:723–750.
[19] Sterling RK, Lissen E, Clumeck N, et al. Development of a simple noninvasive index to predict significant fibrosis in patients with HIV/HCV coinfection. Hepatology 2006;43:1317–1325.
[20] **Kim JH**, **Kim YD**, Lee M, et al. Modified PAGE-B score predicts the risk of hepatocellular carcinoma in Asians with chronic hepatitis B on antiviral therapy. J Hepatol 2018;69:1066–1073.
[21] Giobbie-Hurder A, Gelber RD, Regan MM. Challenges of guarantee-time bias. J Clin Oncol 2013;31:2963–2969.
[22] Yip TC, Wong GL, Chan HL, et al. HBsAg seroclearance further reduces hepatocellular carcinoma risk after complete viral suppression with nucleos(t)ide analogues. J Hepatol 2019;70:361–370.
[23] You SL, Yang HI, Chen CJ. Seropositivity of hepatitis B e antigen and hepatocellular carcinoma. Ann Med 2004;36:215–224.
[24] Choi J, Kim GA, Han S, et al. Earlier alanine aminotransferase normalization during antiviral treatment is independently associated with lower risk of hepatocellular carcinoma in chronic hepatitis B. Am J Gastroenterol 2020;115:406–414.
[25] Liang LY, Wong VW, Toyoda H, et al. Serum hepatitis B core-related antigen predicts hepatocellular carcinoma in hepatitis B e antigen-negative patients. J Gastroenterol 2020;55:899–908.
[26] Papatheodoridis GV, Voulgaris T, Papatheodoridi M, et al. Risk scores for hepatocellular carcinoma in chronic hepatitis B: a promise for precision medicine. Hepatology 2020;72:2197–2205.
[27] **Chun HS**, **Papatheodoridis GV**, Lee M, et al. PAGE-B incorporating moderate HBV DNA levels predicts risk of HCC among patients entering into HBeAg-positive chronic hepatitis B. J Hepatol 2024;80:20–30.
[28] Liaw YF, Lau GK, Kao JH, et al. Hepatitis B e antigen seroconversion: a critical event in chronic hepatitis B virus infection. Dig Dis Sci 2010;55: 2727–2734.
[29] Xing T, Xu H, Cao L, et al. HBeAg seroconversion in HBeAg-positive chronic hepatitis B patients receiving long-term nucleos(t)ide analog treatment: a systematic review and network meta-analysis. PLoS One 2017;12: e0169444.
[30] van Nunen AB, Hansen BE, Suh DJ, et al. Durability of HBeAg seroconversion following antiviral therapy for chronic hepatitis B: relation to type of therapy and pretreatment serum hepatitis B virus DNA and alanine aminotransferase. Gut 2003;52:420–424.
[31] Boni C, Penna A, Bertoletti A, et al. Transient restoration of anti-viral T cell responses induced by lamivudine therapy in chronic hepatitis B. J Hepatol 2003;39:595–605.
[32] Suslov A, Meier MA, Ketterer S, et al. Transition to HBeAg-negative chronic hepatitis B virus infection is associated with reduced cccDNA transcriptional activity. J Hepatol 2021;74:794–800.
[33] Combet C, Bhardwaj N, Hedskog C, et al. Hepatitis B virus genome diversity in adolescents: tenofovir disoproxil fumarate treatment effect and HBeAg serocon version. J Viral Hepat 2021;28:1160–1168.
[34] Buckwold VE, Xu Z, Chen M, et al. Effects of a naturally occurring mutation in the hepatitis B virus basal core promoter on precore gene expression and viral replication. J Virol 1996;70:5845–5851.
[35] Warner BG, Abbott WG, Rodrigo AG. Frequency-dependent selection drives HBeAg seroconversion in chronic hepatitis B virus infection. Evol Med Public Health 2014;2014:1–9.
[36] Chotiyaputta W, Lok AS. Hepatitis B virus variants. Nat Rev Gastroenterol Hepatol 2009;6:453–462.
[37] **Yoo JJ**, **Park SY**, Moon JE, et al. Long-term prognosis and the need for histologic assessment of chronic hepatitis B in the serological immune-tolerant phase. Clin Mol Hepatol 2023;29:482–495.
[38] KASL clinical practice guidelines for management of chronic hepatitis B. Clin Mol Hepatol 2022;28:276–331.
[39] EASL 2017 clinical practice guidelines on the management of hepatitis B virus infection. J Hepatol 2017;67:370–398.
[40] Mak LY, Hui RW, Fung J, et al. The role of different viral biomarkers on the management of chronic hepatitis B. Clin Mol Hepatol 2023;29:263–276.
[41] Choi WM, Kim GA, Choi J, et al. Non-linear association of baseline viral load with on-treatment hepatocellular carcinoma risk in chronic hepatitis B. Gut 2023;73:649–658.
[42] Yang HC, Shih YF, Liu CJ. Viral factors affecting the clinical outcomes of chronic hepatitis B. J Infect Dis 2017;216:S757–S764.
[43] Mommeja-Marin H, Mondou E, Blum MR, et al. Serum HBV DNA as a marker of efficacy during therapy for chronic HBV infection: analysis and review of the literature. Hepatology 2003;37:1309–1319.
[44] Hernán MA, VanderWeele TJ. Compound treatments and transportability of causal inference. Epidemiology 2011;22:368–377.
[45] Austin PC, Stuart EA. Moving towards best practice when using inverse probability of treatment weighting (IPTW) using the propensity score to estimate causal treatment effects in observational studies. Stat Med 2015;34:3661–3679.
[46] Chesnaye NC, Stel VS, Tripepi G, et al. An introduction to inverse probability of treatment weighting in observational research. Clin Kidney J 2022;15: 14–20.
[47] Concato J, Shah N, Horwitz RI. Randomized, controlled trials, observational studies, and the hierarchy of research designs. N Engl J Med 2000;342: 1887–1892.
[48] Kim H, Jee YM, Song BC, et al. Molecular epidemiology of hepatitis B virus (HBV) genotypes and serotypes in patients with chronic HBV infection in Korea. Intervirology 2007;50:52–57.
[49] Chan HL, Hui AY, Wong ML, et al. Genotype C hepatitis B virus infection is associated with an increased risk of hepatocellular carcinoma. Gut 2004;53: 1494–1498.
[50] Chu CJ, Hussain M, Lok AS. Hepatitis B virus genotype B is associated with earlier HBeAg seroconversion compared with hepatitis B virus genotype C. Gastroenterology 2002;122:1756–1762.

Keywords: Nucleos(t)ide analogue; Entecavir; Tenofovir; Liver cancer; Time-varying effects.
Received 12 December 2023; received in revised form 26 March 2024; accepted 2 April 2024; Available online 8 April 2024

Journal of Hepatology, Volume *6*

Supplemental information

Lack of association between early on-treatment HBeAg seroclearance and development of hepatocellular carcinoma or decompensated cirrhosis

Hyunjae Shin, Won-Mook Choi, Seung Up Kim, Yunmi Ko, Youngsu Park, Jeayeon Park, Moon Haeng Hur, Min Kyung Park, Yun Bin Lee, Yoon Jun Kim, Jung-Hwan Yoon, Jeong-Hoon Lee, and Fabien Zoulim

Lack of association between early on-treatment HBeAg seroclearance and development of hepatocellular carcinoma or decompensated cirrhosis

Hyunjae Shin, Won-Mook Choi, Seung Up Kim, Yunmi Ko, Youngsu Park, Jeayeon Park, Moon Haeng Hur, Min Kyung Park, Yun Bin Lee, Yoon Jun Kim, Jung-Hwan Yoon, Jeong-Hoon Lee, Fabien Zoulim

Table of contents

Fig. S1……………………………………………………………………………2
Fig. S2……………………………………………………………………………3
Fig. S3……………………………………………………………………………4
Fig. S4……………………………………………………………………………5
Fig. S5……………………………………………………………………………6
Fig. S6……………………………………………………………………………7
Fig. S7……………………………………………………………………………8
Table S1…………………………………………………………………………..9
Table S2…………………………………………………………………………10
Table S3…………………………………………………………………………11
Table S4…………………………………………………………………………12
Table S5…………………………………………………………………………13
Table S6…………………………………………………………………………14
Table S7…………………………………………………………………………15
Table S8…………………………………………………………………………16
Table S9………………………………………………………………………......17

Supplementary Figures

Fig. S1. Cumulative incidence rate of HBeAg-seroclearance.

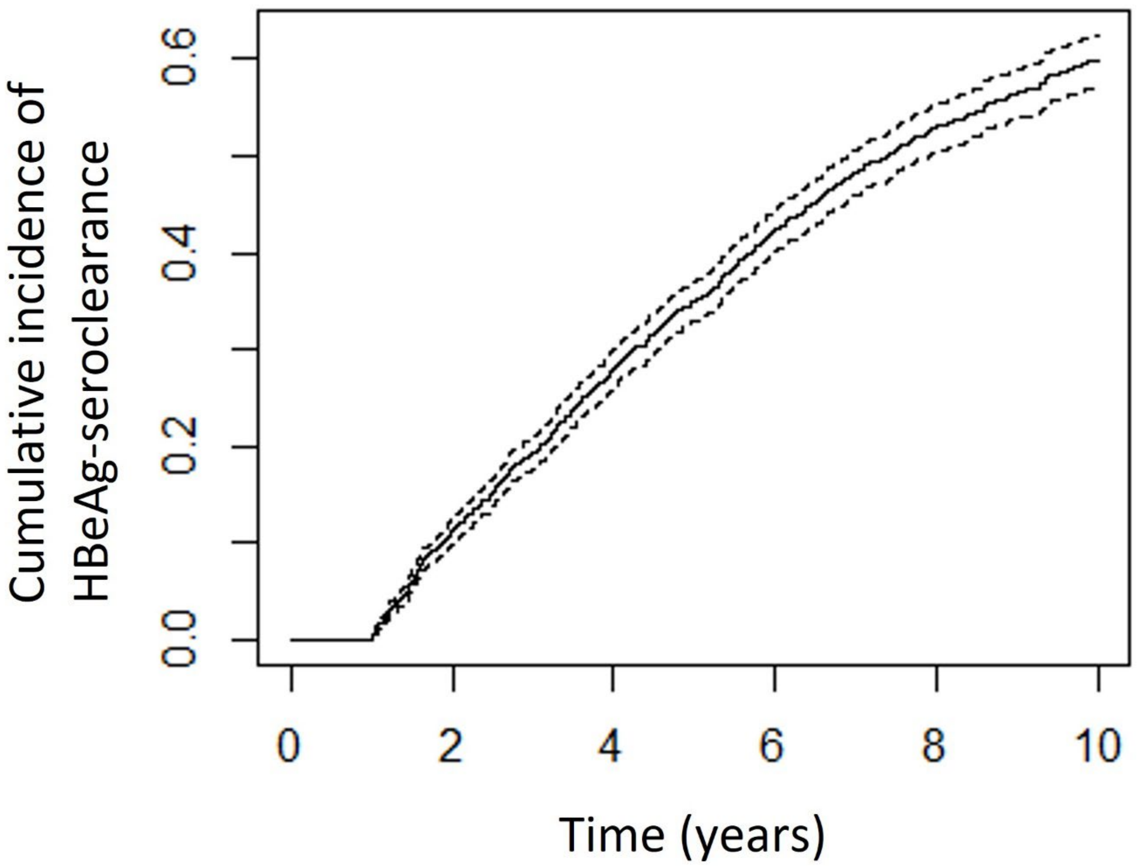

HBeAg, hepatitis B virus envelope antigen

Fig. S2. Risk of (A) decompensated liver cirrhosis, and (B) liver-related outcomes according to HBeAg-seroclearance at 36 months after initiation of antiviral treatment. Weight analysis balanced by inverse probability of treatment weighting.

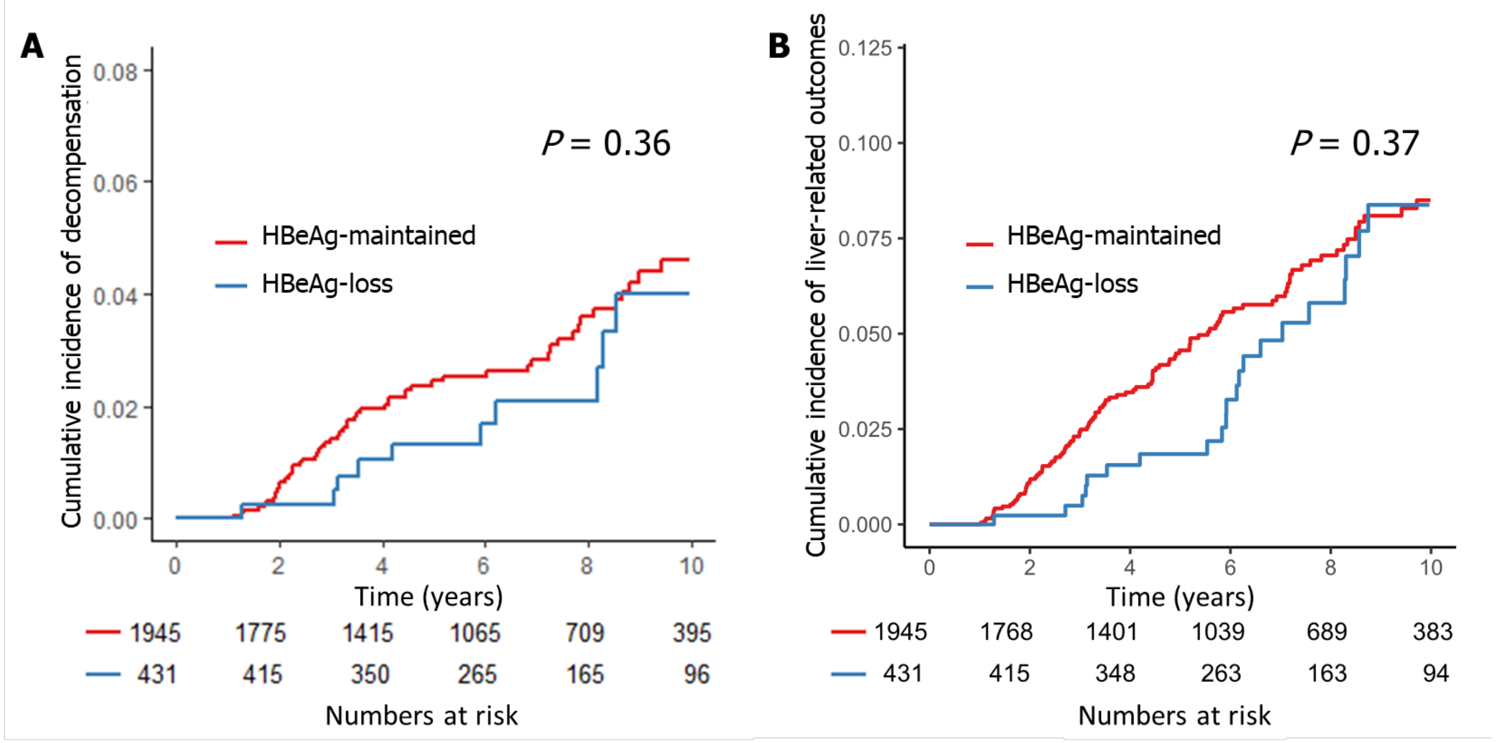

HBeAg, hepatitis B virus envelope antigen

The cumulative incidences of HCC were compared by Kaplan-Meier curves, and *P* values were derived from log-rank test.

Fig. S3. Risk of HCC according to HBeAg-seroclearance at 60 months after initiation of antiviral treatment. (A) Unweighted analysis. (B) Weight analysis balanced by inverse probability of treatment weighting.

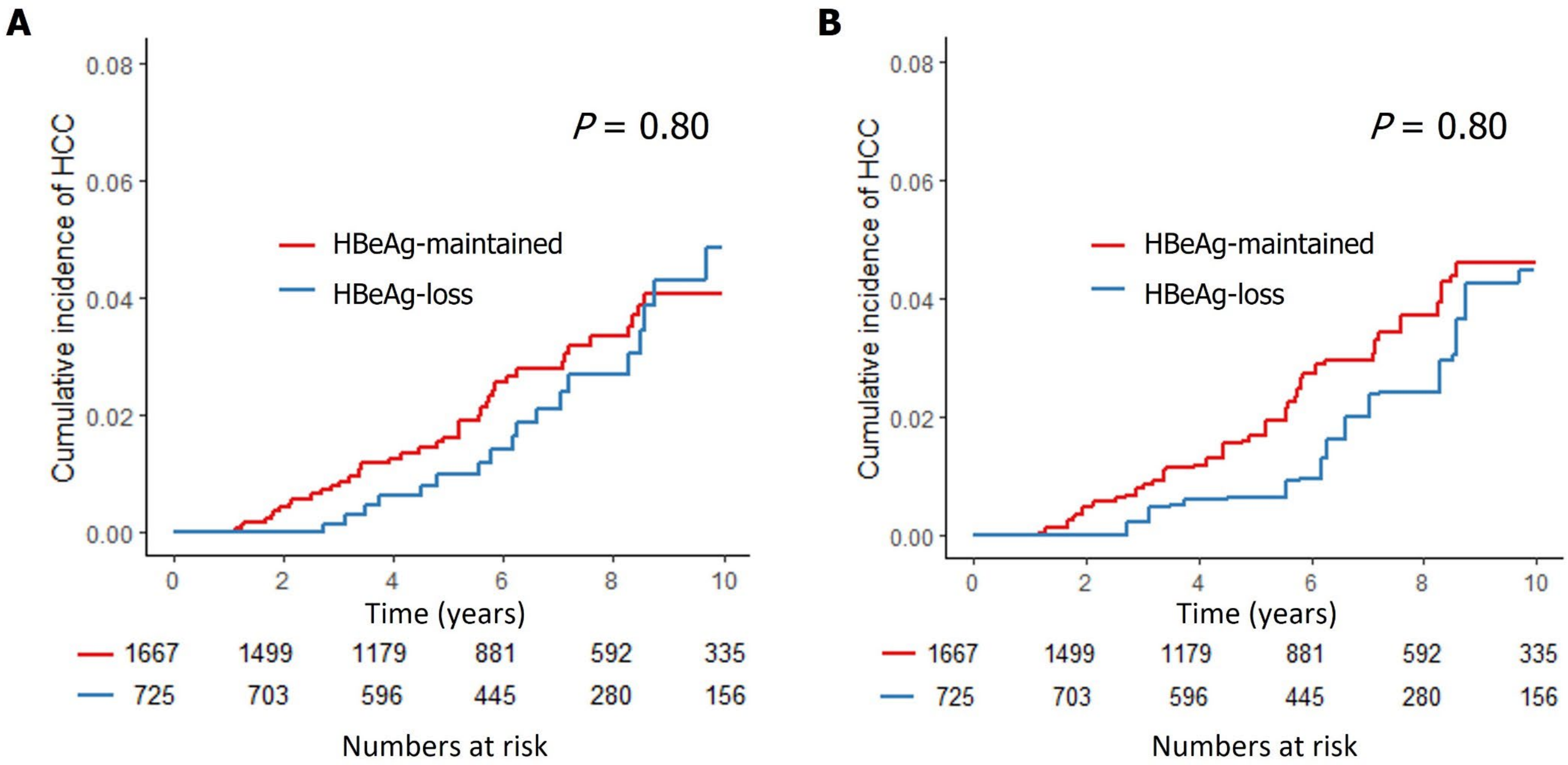

HCC, hepatocellular carcinoma; HBeAg, hepatitis B virus envelope antigen

The cumulative incidences of HCC were compared by Kaplan-Meier curves, and *P* values were derived from log-rank test.

Fig. S4. Risk of HCC according to HBeAg-seroclearance at 36 months after initiation of antiviral treatment. (A) Unweighted analysis. (B) Weight analysis balanced by inverse probability of treatment weighting in the subgroup of platelet count of more than 150×10^3/μL.

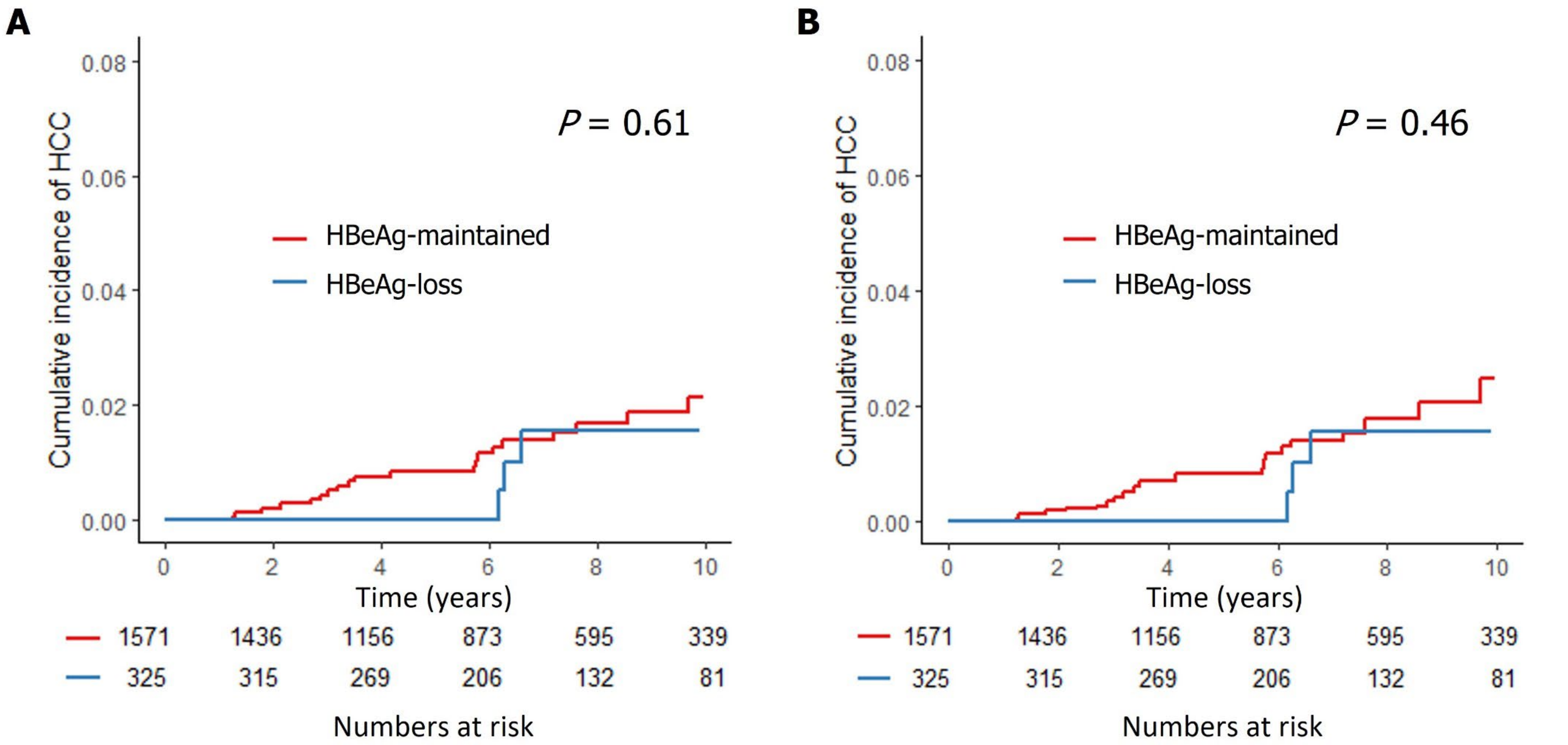

HCC, hepatocellular carcinoma; HBeAg, hepatitis B virus envelope antigen

The cumulative incidences of HCC were compared by Kaplan-Meier curves, and *P* values were derived from log-rank test.

Fig. S5. Risk of HCC according to HBeAg-seroclearance at 36 months after initiation of antiviral treatment in FIB-4 stratified groups. (A) high FIB-4 group (i.e., ≥ 2.0), and (B) low FIB-4 group (i.e., < 2.0).

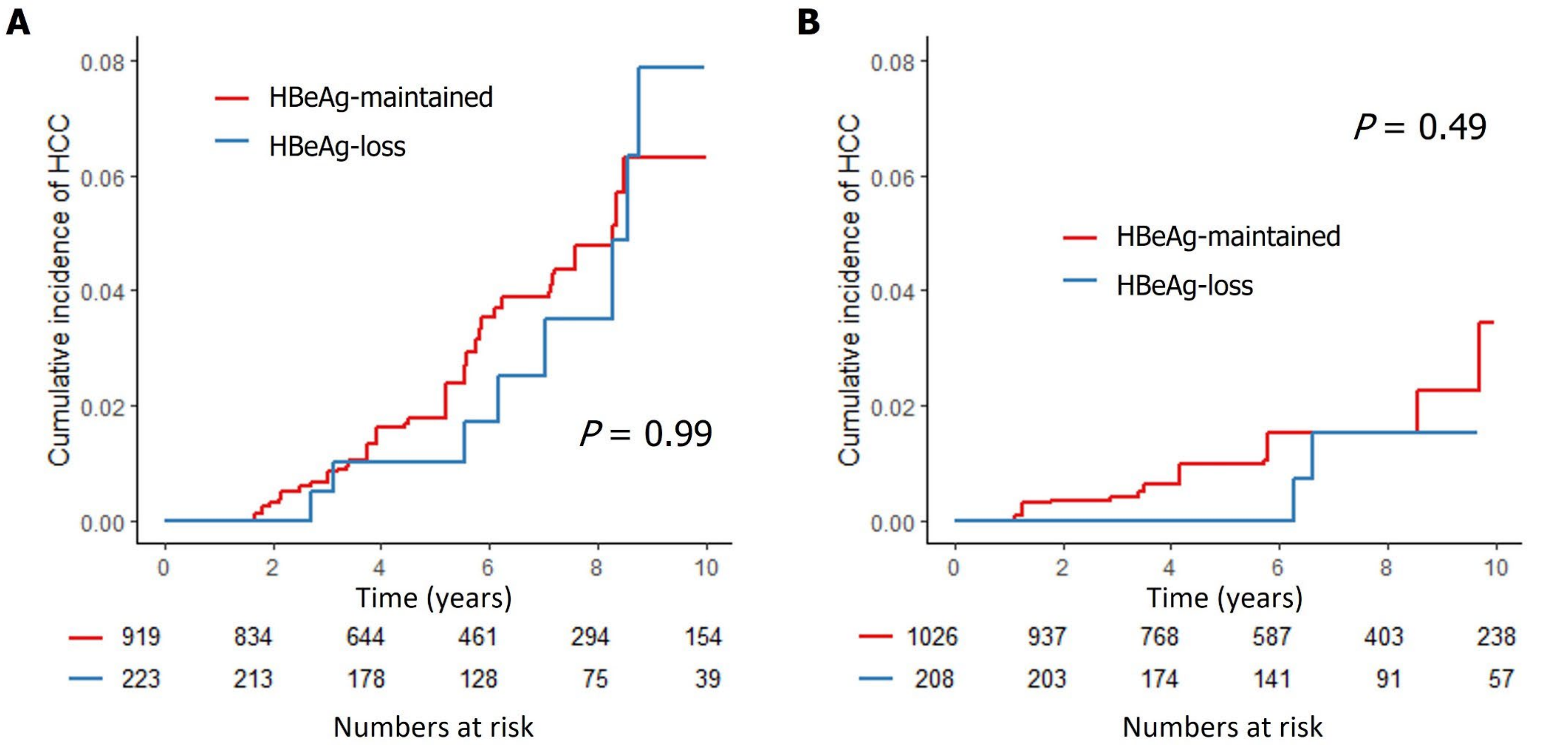

HCC, hepatocellular carcinoma; HBeAg, hepatitis B virus envelope antigen; FIB-4, fibrosis-4

The cumulative incidences of HCC were compared by Kaplan-Meier curves, and *P* values were derived from log-rank test.

Fig. S6. Risk of HCC according to HBeAg-seroclearance at 36 months after initiation of antiviral treatment in age stratified groups. (A) older age group (i.e., ≥ 45), and (B) younger age group (i.e., < 45).

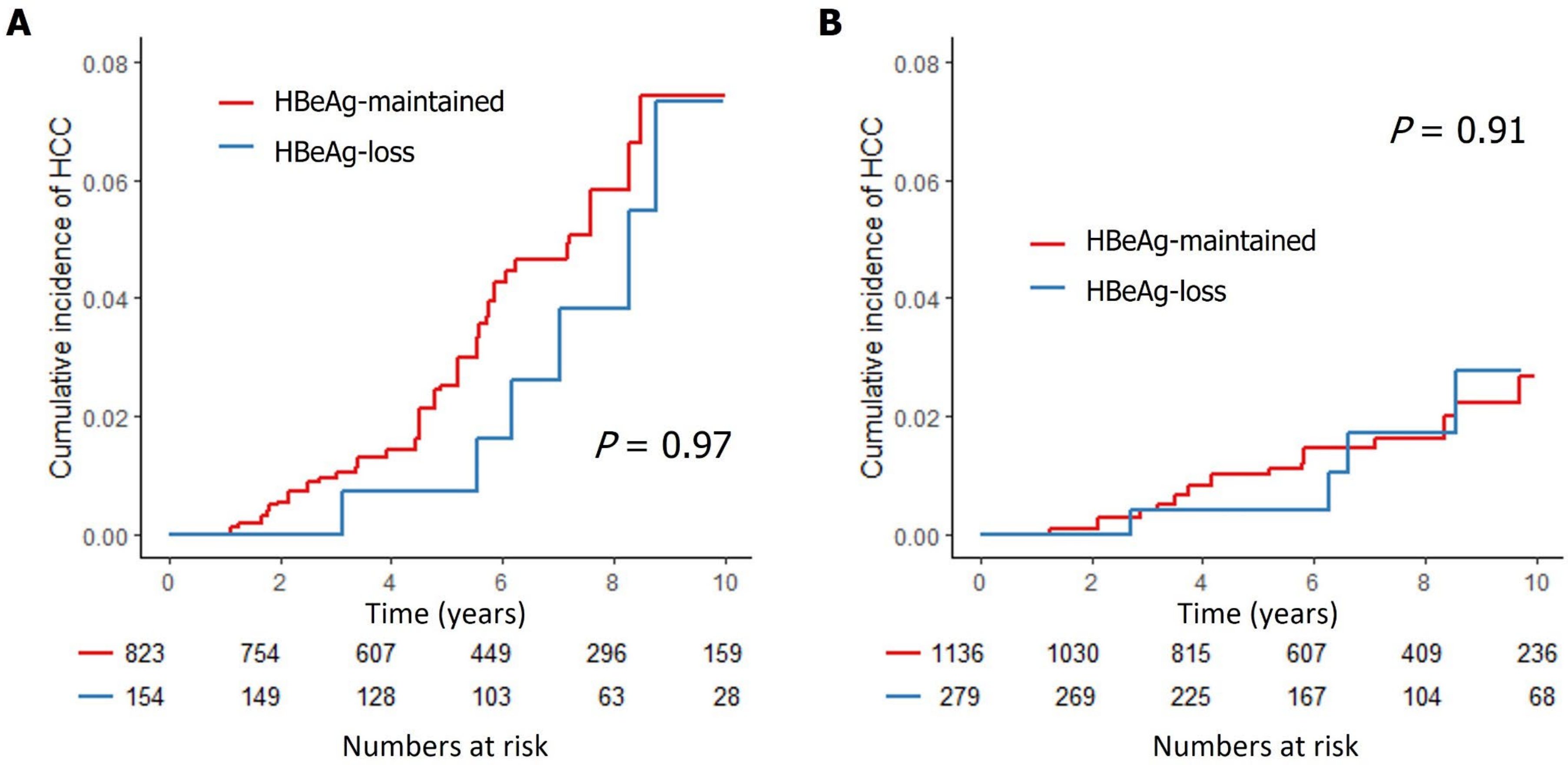

HCC, hepatocellular carcinoma; HBeAg, hepatitis B virus envelope antigen

The cumulative incidences of HCC were compared by Kaplan-Meier curves, and *P* values were derived from log-rank test.

Fig. S7. Risk of HCC according to HBV DNA suppression (i.e., serum HBV DNA ≤ 2,000 U/mL) at 4 months and baseline HBV DNA level after initiation of antiviral treatment.

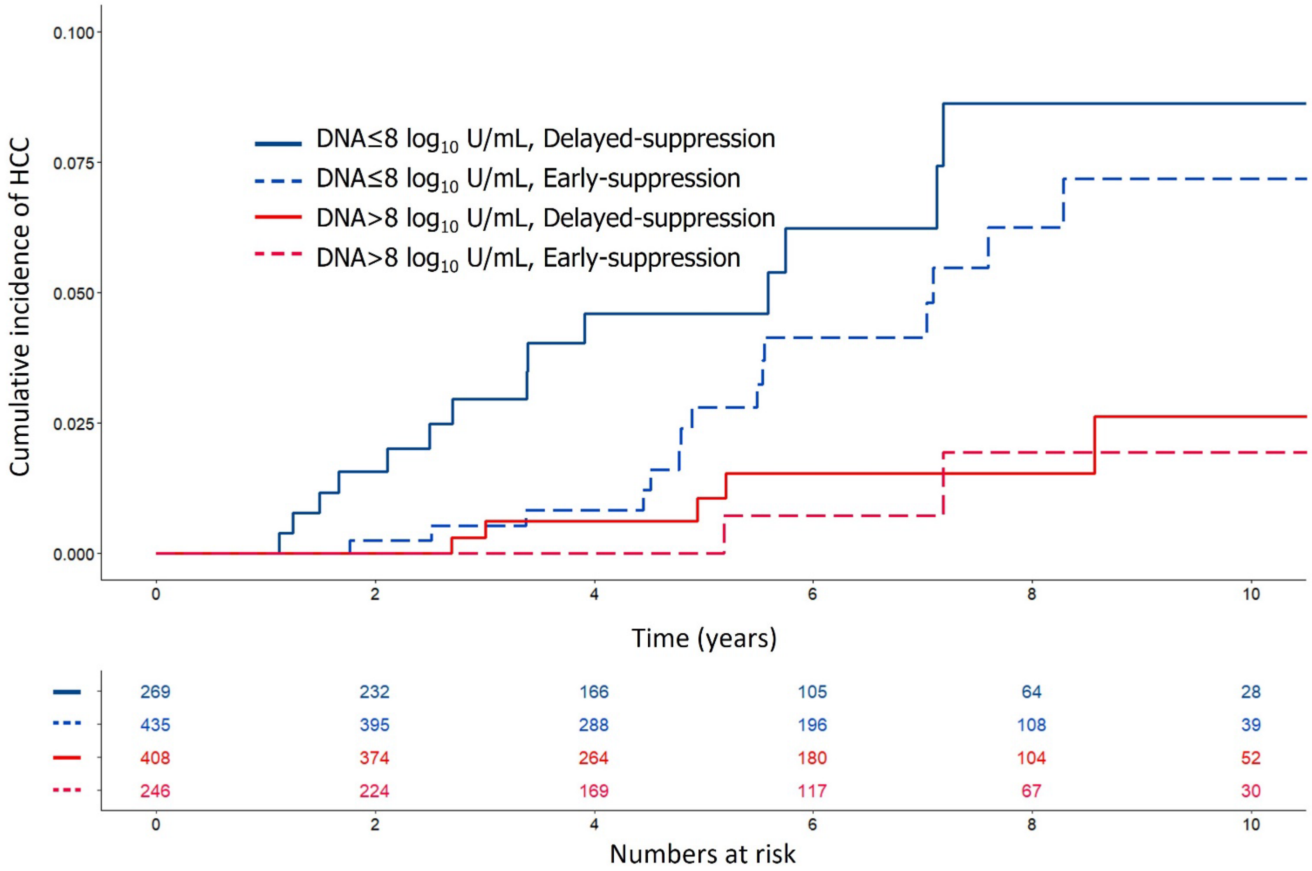

HCC, hepatocellular carcinoma

The cumulative incidences of HCC were compared by Kaplan-Meier curves, and *P* values were derived from log-rank test.

Supplementary Tables

Table S1. Baseline characteristics of the subgroup of platelet count of more than $150\times10^3/\mu L$, and the HBeAg-maintained and HBeAg-loss groups before and after inverse probability of treatment weighting.

Characteristics	Platelet > 150K	Before IPTW HBeAg-seroclearance within 36 months				After IPTW HBeAg-seroclearance within 36 months			
		**HBeAg-maintained**	**HBeAg-loss**	***P***	**SMD**	**HBeAg-maintained**	**HBeAg-loss**	***P***	**SMD**
	n = 1,896	*n* = 1,571	*n* = 325			*n* = 1,571	*n* = 325		
Age, years	40 (32–48)	40 (32–49)	37 (31–46)	<0.001	0.205	38 (31–47)	39 (32–48)	0.23	0.073
Sex, male, %	1,202 (63.4%)	1,010 (64.3%)	192 (59.1%)	0.08	0.107	998 (63.5%)	192 (59.1%)	0.14	0.090
Antiviral, %				0.50	0.043			0.53	0.039
Entecavir	1,112 (59.2%)	924 (58.8%)	198 (60.9%)			928 (59.1%)	198 (60.9%)		
TDF	774 (40.8%)	647 (41.2%)	127 (39.1%)			643 (40.9%)	127 (39.1%)		
Platelet count, $10^3/\mu L$	203 (177–238)	204 (177–239)	200 (175–232)	0.19	0.053	202 (176–235)	200 (174–232)	0.68	0.007
Albumin, g/dL	4.1 (3.8–4.3)	4.1 (3.8–4.3)	4.1 (3.8–4.3)	0.09	0.105	4.1 (3.8–4.3)	4.1 (3.8–4.3)	0.47	0.037
AST, U/L	98 (63–179)	96 (60–167)	111 (75–234)	<0.001	0.131	111 (72–211)	111 (75–234)	0.31	0.005
ALT, U/L	130 (84–247)	125 (84–236)	157 (87–305)	<0.001	0.201	149 (90–294)	157 (86–305)	0.61	0.035
HBV DNA, $\log_{10}$ U/mL	8.0 (7.0–8.2)	8.0 (7.1–8.3)	7.9 (6.9–8.2)	<0.001	0.111	8.0 (6.9–8.2)	7.9 (6.9–8.2)	0.64	0.010
Total bilirubin, mg/dL	0.9 (0.7–1.2)	0.9 (0.7–1.2)	0.9 (0.7–1.2)	0.37	0.099	0.9 (0.7–1.2)	0.9 (0.7–1.2)	0.92	0.033
Creatinine, mg/dL	0.9 (0.7–1.0)	0.9 (0.7–1.0)	0.9 (0.8–1.0)	0.57	0.021	0.9 (0.8–1.0)	0.9 (0.8–1.0)	0.89	0.013
FIB-4	1.7 (1.1–2.6)	1.7 (1.1–2.5)	1.7 (1.1–2.8)	0.16	0.095	1.8 (1.1–2.8)	1.7 (1.1–2.8)	0.93	0.015
mPAGE-B	8 (6–10)	8 (6–10)	8 (5–10)	0.047	0.120	8 (5–10)	8 (5–10)	0.52	0.041

IPTW, inverse probability of treatment weighting; HBeAg, hepatitis B virus envelope antigen; SMD, standardized mean difference; TDF, tenofovir disoproxil fumarate; AST, aspartate aminotransferase; ALT, alanine aminotransferase; FIB-4, Fibrosis-4; mPAGE-B, modified PAGE-B

All variables were compared between the HBeAg-loss and HBeAg-maintained groups before and after IPTW. Sex, and the type of antiviral treatment were analyzed by Fisher's exact test. Other variables were analyzed by Kruskal–Wallis test and expressed in median (IQR).

Table S2. Baseline characteristics of the stratified subgroup of the FIB-4 index ≥ 2.0, and the HBeAg-maintained and HBeAg-loss groups before and after inverse probability of treatment weighting.

Characteristics	FIB-4 ≥ 2.0	Before IPTW HBeAg-seroclearance within 36 months				After IPTW HBeAg-seroclearance within 36 months			
		HBeAg-maintained	HBeAg-loss	*P*	SMD	HBeAg-maintained	HBeAg-loss	*P*	SMD
	n = 1,142	*n* = 919	*n* = 223			*n* = 919	*n* = 223		
Age, years	47 (38–55)	48 (39–55)	45 (36–53)	0.02	0.165	46 (37–54)	45 (36–53)	0.70	0.023
Sex, male, %	654 (57.3%)	537 (58.4%)	117 (52.5%)	0.11	0.120	510 (55.5%)	117 (52.5%)	0.43	0.060
Antiviral, %									
Entecavir	673 (58.9%)	540 (58.8%)	133 (59.6%)	0.82	0.018	541 (58.9%)	133 (59.6%)	0.85	0.014
TDF	469 (41.1%)	379 (41.2%)	90 (40.4%)			378 (41.1%)	90 (40.4%)		
Platelet count, 10^3/µL	166 (134–198)	167 (135–199)	162 (131–193)	0.18	0.084	164 (134–195)	161 (131–192)	0.63	0.023
Albumin, g/dL	3.9 (3.6–4.2)	3.9 (3.6–4.2)	3.9 (3.6–4.2)	0.26	0.064	3.9 (3.6–4.2)	3.9 (3.6–4.2)	0.32	0.063
AST, U/L	150 (90–273)	142 (88–264)	170 (96–295)	0.03	0.061	170 (97–300)	170 (95–290)	0.88	0.031
ALT, U/L	139 (82–292)	134 (82–287)	149 (84–323)	0.06	0.107	154 (88–333)	148 (84–322)	0.96	0.001
HBV DNA, $\log_{10}$ U/mL	8.0 (6.9–8.2)	8.0 (6.9–8.2)	7.8 (6.9–8.2)	0.19	0.013	8.0 (7.0–8.2)	7.8 (6.9–8.2)	0.14	0.049
Total bilirubin, mg/dL	1.0 (0.8–1.3)	1.0 (0.8–1.3)	1.0 (0.8–1.4)	0.20	0.041	1.0 (0.8–1.4)	1.0 (0.8–1.4)	0.57	0.028
Creatinine, mg/dL	0.8 (0.7–1.0)	0.9 (0.7–1.0)	0.8 (0.7–1.0)	0.81	0.105	0.8 (0.7 -1.0)	0.8 (0.7 -1.0)	1.00	0.090
FIB-4	3.4 (2.5–4.7)	3.3 (2.5–4.7)	3.6 (2.6–4.9)	0.21	0.079	3.5 (2.6–4.8)	3.6 (2.6–4.9)	0.83	0.039
mPAGE-B	10 (8–12)	10 (8–12)	10 (8–12)	0.17	0.099	10 (8–12)	10 (8–12)	0.93	0.007

IPTW, inverse probability of treatment weighting; HBeAg, hepatitis B virus envelope antigen; SMD, standardized mean difference; TDF, tenofovir disoproxil fumarate; AST, aspartate aminotransferase; ALT, alanine aminotransferase; FIB-4, Fibrosis-4; mPAGE-B, modified PAGE-B

All variables were compared between the HBeAg-loss and HBeAg-maintained groups before and after IPTW. Sex, and the type of antiviral treatment were analyzed by Fisher's exact test. Other variables were analyzed by Kruskal–Wallis test and expressed in median (IQR).

Table S3. Baseline characteristics of the stratified subgroup of the FIB-4 index < 2.0, and the HBeAg-maintained and HBeAg-loss groups before and after inverse probability of treatment weighting.

Characteristics	FIB-4 < 2.0	Before IPTW HBeAg-seroclearance within 36 months				After IPTW HBeAg-seroclearance within 36 months			
		HBeAg-maintained	HBeAg-loss	*P*	SMD	HBeAg-maintained	HBeAg-loss	*P*	SMD
	n = 1,234	*n* = 1,026	*n* = 208			*n* = 1,026	*n* = 208		
Age, years	36 (30–44)	37 (30–45)	33 (29–41)	<0.001	0.307	34 (29–42)	33 (29–41)	0.41	0.078
Sex, male, %	829 (67.2%)	690 (67.3%)	138 (66.3%)	0.81	0.021	682 (66.5%)	138 (66.2%)	0.93	0.007
Antiviral, %				0.35	0.076			0.60	0.041
Entecavir	727 (58.9%)	598 (58.3%)	129 (62%)			614 (59.8%)	129 (61.8%)		
TDF	507 (41.1%)	428 (41.7%)	79 (38%)			412 (40.2%)	79 (38.2%)		
Platelet count, 10^3/μL	211 (182- 246)	212 (183–246)	203 (177–243)	0.16	0.069	206 (176–243)	203(177–242)	0.84	0.011
Albumin, g/dL	4.1 (3.9–4.4)	4.1 (3.9–4.4)	4.1 (3.9–4.3)	0.20	0.107	4.1 (3.9–4.4)	4.1 (3.9–4.3)	0.32	0.071
AST, U/L	73 (50–108)	70 (49–104)	82 (60–118)	<0.001	0.237	78 (52–127)	81 (59–118)	0.27	0.044
ALT, U/L	117 (83–198)	115 (83–191)	137 (83–247)	0.02	0.219	130 (85–237)	136 (83–247)	0.86	0.041
HBV DNA, $\log_{10}$ U/mL	8.0 (6.8–8.3)	8.0 (6.9–8.4)	7.9 (6.6–8.2)	<0.001	0.175	7.9 (6.7–8.2)	7.9 (6.6–8.2)	0.49	0.047
Total bilirubin, mg/dL	0.9 (0.7–1.1)	0.9 (0.7–1.1)	0.9 (0.7–1.1)	0.35	0.161	0.9 (0.7–1.1)	0.9 (0.7–1.1)	0.59	0.145
Creatinine, mg/dL	0.9 (0.8–1.0)	0.9 (0.8–1.0)	0.9 (0.8–1.0)	0.79	0.090	0.9 (0.8–1.0)	0.9 (0.8–1.0)	0.83	0.105
FIB-4	1.2 (0.9–1.6)	1.2 (0.9–1.6)	1.2 (0.9–1.6)	0.60	0.047	1.2 (0.9–1.6)	1.2 (0.9–1.6)	0.70	0.031
mPAGE-B	7 (5–9)	7 (5–9)	7 (5–9)	0.19	0.122	7 (5–9)	7 (5–9)	0.87	0.004

IPTW, inverse probability of treatment weighting; HBeAg, hepatitis B virus envelope antigen; SMD, standardized mean difference; TDF, tenofovir disoproxil fumarate; AST, aspartate aminotransferase; ALT, alanine aminotransferase; FIB-4, Fibrosis-4; mPAGE-B, modified PAGE-B

All variables were compared between the HBeAg-loss and HBeAg-maintained groups before and after IPTW. Sex, and the type of antiviral treatment were analyzed by Fisher’s exact test. Other variables were analyzed by Kruskal–Wallis test and expressed in median (IQR).

Table S4. Baseline characteristics of the stratified subgroup of age ≥ 45, and the HBeAg-maintained and HBeAg-loss groups before and after inverse probability of treatment weighting.

Characteristics	Age ≥ 45	Before IPTW HBeAg-seroclearance within 36 months				After IPTW HBeAg-seroclearance within 36 months			
		HBeAg-maintained	HBeAg-loss	*P*	SMD	HBeAg-maintained	HBeAg-loss	*P*	SMD
	n = 977	*n* = 823	*n* = 154			*n* = 823	*n* = 154		
Age, years	52 (48–57)	52 (48–57)	51 (47–56)	0.10	0.095	52 (48–57)	51 (47–56)	0.28	0.067
Sex, male, %	514 (56.2%)	432 (52.5%)	82 (53.2%)	0.93	0.015	432 (52.5%)	82 (53.2%)	0.88	0.014
Antiviral, %									
Entecavir	592 (60.6%)	438 (53.2%)	96 (62.3%)	0.65	0.043	494 (60.0%)	96 (62.3%)	0.59	0.048
TDF	385 (39.4%)	385 (46.8%)	58 (37.7%)			329 (40.0%)	58 (37.7%)		
Platelet count, $10^3/\mu L$	173 (141–211)	176 (145–212)	161 (132–197)	0.01	0.145	164 (134–200)	160 (132–197)	0.65	0.006
Albumin, g/dL	3.9 (3.6–4.2)	3.9 (3.6–4.2)	3.9 (3.6–4.1)	0.15	0.102	3.9 (3.6–4.2)	3.9 (3.6–4.1)	0.41	0.076
AST, U/L	90 (62–144)	89 (62–136)	99 (66–166)	0.08	0.095	95 (65–160)	97 (66–165)	0.71	0.007
ALT, U/L	106 (72–180)	106 (72- 177)	103 (73–195)	0.97	0.004	107 (72–187)	102 (73–194)	0.84	0.015
HBV DNA, log_{10} U/mL	8.0 (6.6–8.2)	8.0 (6.6–8.2)	7.8 (6.4–8.2)	0.05	0.139	7.9 (6.6–8.2)	7.8 (6.3–8.2)	0.34	0.072
Total bilirubin, mg/dL	0.9 (0.7–1.2)	0.9 (0.7–1.2)	0.9 (0.7–1.2)	0.28	0.011	0.9 (0.7–1.2)	0.9 (0.7–1.2)	0.51	0.032
Creatinine, mg/dL	0.8 (0.7–1.0)	0.8 (0.7–1.0)	0.8 (0.7–0.9)	1.00	0.141	0.8 (0.7–1.0)	0.8 (0.7–0.9)	0.98	0.110
FIB-4	2.7 (1.8–4.2)	2.6 (1.8–4.2)	3.1 (2.1–4.7)	0.01	0.223	3.1 (2.0–4.6)	3.1 (2.1–4.7)	0.73	0.090
mPAGE-B	11 (10–13)	11 (10–13)	11 (10–13)	0.43	0.094	11 (10–13)	11 (10–13)	0.91	0.024

IPTW, inverse probability of treatment weighting; HBeAg, hepatitis B virus envelope antigen; SMD, standardized mean difference; TDF, tenofovir disoproxil fumarate; AST, aspartate aminotransferase; ALT, alanine aminotransferase; FIB-4, Fibrosis-4; mPAGE-B, modified PAGE-B

All variables were compared between the HBeAg-loss and HBeAg-maintained groups before and after IPTW. Sex, and the type of antiviral treatment were analyzed by Fisher's exact test. Other variables were analyzed by Kruskal–Wallis test and expressed in median (IQR).

Table S5. Baseline characteristics of the stratified subgroup of age < 45, and the HBeAg-maintained and HBeAg-loss groups before and after inverse probability of treatment weighting.

Characteristics	Age < 45	Before IPTW HBeAg-seroclearance within 36 months				After IPTW HBeAg-seroclearance within 36 months			
		HBeAg-maintained	HBeAg-loss	*P*	SMD	HBeAg-maintained	HBeAg-loss	*P*	SMD
	n = 1,415	*n* = 1,136	*n* = 279			*n* = 1,136	*n* = 279		
Age, years	34 (29–39)	34 (29–39)	33 (29–38)	0.10	0.122	34 (29–39)	33 (29–38)	0.17	0.099
Sex, male, %	979 (69.2 %)	805 (70.9%)	174 (62.4%)	0.01	0.181	779 (68.6%)	96 (62.4%)	0.05	0.131
Antiviral, %									
Entecavir	822 (58.1%)	655 (57.7%)	167 (59.9%)	0.54	0.045	662 (58.3%)	167 (59.9%)	0.64	0.031
TDF	593 (41.9%)	481 (42.3%)	112 (40.1%)			474 (41.7%)	112 (40.1%)		
Platelet count, 10^3/μL	200 (168–237)	202 (169–240)	194 (166–230)	0.04	0.138	198 (166–233)	194 (166–230)	0.40	0.097
Albumin, g/dL	4.1 (3.9–4.4)	4.1 (3.9–4.4)	4.1 (3.8–4.3)	0.02	0.160	4.1 (3.8–4.3)	4.1 (3.8–4.3)	0.41	0.031
AST, U/L	105 (64–206)	101 (60–195)	118 (76–246)	<0.001	0.092	120 (73–228)	118 (76–246)	0.39	<0.001
ALT, U/L	144 (88–288)	138 (87–275)	176 (96–362)	<0.001	0.182	163 (94–339)	176 (95–361)	0.35	0.048
HBV DNA, $\log_{10}$ U/mL	8.0 (7.0–8.2)	8.0 (7.1–8.2)	7.9 (7.0–8.2)	0.001	0.086	7.9 (6.9–8.2)	7.9 (6.9–8.2)	0.42	0.015
Total bilirubin, mg/dL	0.9 (0.7–1.2)	0.9 (0.7–1.2)	1.0 (0.7–1.3)	0.17	0.131	0.9 (0.7–1.3)	0.9 (0.7–1.3)	0.85	0.043
Creatinine, mg/dL	0.9 (0.8–1.0)	0.9 (0.8–1.0)	0.9 (0.8–1.0)	0.87	0.044	0.9 (0.7–1.0)	0.9 (0.8–1.0)	0.62	0.022
FIB-4	1.5 (0.9–2.5)	1.5 (0.9–2.4)	1.6 (1.1–2.7)	0.01	0.066	1.6 (1.1–2.7)	1.6 (1.1–2.7)	0.85	0.023
mPAGE-B	7 (5–9)	7 (5–9)	7 (5–9)	0.99	0.003	7 (5–9)	7 (5–9)	0.66	0.018

IPTW, inverse probability of treatment weighting; HBeAg, hepatitis B virus envelope antigen; SMD, standardized mean difference; TDF, tenofovir disoproxil fumarate; AST, aspartate aminotransferase; ALT, alanine aminotransferase; FIB-4, Fibrosis-4; mPAGE-B, modified PAGE-B

All variables were compared between the HBeAg-loss and HBeAg-maintained groups before and after IPTW. Sex, and the type of antiviral treatment were analyzed by Fisher's exact test. Other variables were analyzed by Kruskal–Wallis test and expressed in median (IQR).

Table S6. Univariable and multivariable Cox regression analyses with stratification by the timing of HBeAg-seroclearance: 1–3, 3–5, 5–7, 7–10, ≥10 years.

Characteristics	Univariable analysis			Multivariable analysis		
	**HR[1]**	**95% CI[1]**	***P***	**HR[1]**	**95% CI[1]**	***P***
Time to HBeAg-seroclearance, years						
≥ 10	1	[Reference]				
1–3	1.22	0.56, 2.68	0.62	1.05	0.46, 2.39	0.97
3–5	1.83	0.86, 3.93	0.12	1.64	0.72, 3.74	0.24
5–7	0.72	0.28, 1.86	0.49	0.74	0.28, 2.01	0.56
7–10	1.16	0.53, 2.56	0.71	1.28	0.56, 2.93	0.55
Sex, male	2.18	1.18, 4.01	0.012	3.14	1.66, 5.92	<0.001
Age, years	1.06	1.04, 1.09	<0.001	1.04	1.01, 1.07	<0.001
Platelet count, 10^3/μL						
> 250	1	[Reference]				
150–250	5.52	0.75, 40.7	0.94	4.43	0.60, 32.8	0.15
< 150	29.1	3.99, 212	<0.001	16.4	2.19, 123	0.01
ALT						
≥ 2 x ULN[1]	1	[Reference]				
1–2 x ULN[1]	3.5	2.13, 5.76	<0.001	2.71	1.56, 4.70	<0.001
HBV DNA, $\log_{10}$ U/mL	0.74	0.64, 0.85	<0.001	0.85	0.72, 0.99	0.04
FIB-4	1.14	1.08, 1.20	<0.001	1.05	0.97, 1.14	0.20
[1] 40 U/L						

HCC, hepatocellular carcinoma; HR, hazard ratio; CI, confidence interval; aHR, adjusted hazard ratio; HBeAg, hepatitis B virus envelope antigen; ALT, alanine aminotransferase; ULN, upper limit of normal; FIB-4, Fibrosis-4

Multivariable Cox regression analysis was performed on covariates associated with HCC occurrence identified in univariable analysis. The results were expressed in terms of HR, 95% CI, and *P* values. This analysis included a time-dependent covariate, HBeAg seroclearance, which was the variable of interest.

Table S7. Univariable and multivariable time-dependent Cox regression analyses for the predictive factors of decompensated liver cirrhosis occurrence

Characteristics	Univariable analysis			Multivariable analysis		
	**HR**	**95% CI**	***P***	**aHR**	**95% CI**	***P***
On-treatment variables						
HBeAg-seroclearance	0.83	0.61, 1.91	0.61	0.87	0.68, 1.75	0.54
Baseline variables						
Sex, male	1.78	1.04, 3.75	0.02	2.49	1.26, 4.80	0.001
Age, years	1.07	1.03, 1.11	<0.001	1.05	1.03, 1.07	<0.001
Platelet count, 10^3/μL						
>250	1	[Reference]				
150–250	4.59	0.78, 34.5	0.18	3.54	0.47, 28.1	0.30
<150	41.7	5.60, 301	<0.001	22.5	4.12, 194	0.001
ALT						
≥2 x ULN[1]	1	[Reference]				
1–2 x ULN	2.50	1.82, 5.47	<0.001	2.36	1.60, 4.98	<0.001
HBV DNA, $\log_{10}$ U/mL	0.69	0.54, 0.81	<0.001	0.78	0.68, 0.92	0.001
FIB-4	1.04	1.01, 1.09	0.003	1.02	0.96, 1.07	0.15

[1] The upper limit of normal ALT was defined as 40 IU/L.

aHR, adjusted hazard ratio; ALT, alanine aminotransferase; CI, confidence interval; FIB-4, fibrosis-4 ; HBeAg, hepatitis B virus envelope antigen; HBV, hepatitis B virus; HR, hazard ratio

Multivariable Cox regression analysis was performed on covariates associated with HCC occurrence identified in univariable analysis. The results were expressed in terms of HR, 95% CI, and *P* values. This analysis included a time-dependent covariate, HBeAg seroclearance, which was the variable of interest.

Table S8. Summarized result of HCC occurence by time-dependent Cox analyses with multiple landmarks as 24, 36, 48, 60 months after initiation of antiviral treatment.

Cox proportional hazard analysis with IPTW-balanced groups			
**HBeAg-seroclearance within 36 months from initiation of antivirals**			
	n	HR (95% CI)	*P*
HBeAg-loss	434	0.89 (0.47–1.68)	0.72
HBeAg-maintained	1,948		
**HBeAg-seroclearance within 60 months from initiation of antivirals**			
	n	HR (95% CI)	*P*
HBeAg-loss	725	0.85 (0.50–1.46)	0.57
HBeAg-maintained	1,657		
**Time-dependent Cox regression analysis**			
	n	HR (95% CI)	*P*
HBeAg-loss	1,077	1.10 (0.64–1.92)	0.70
HBeAg-maintained	1,305		
**Time-dependent Cox regression analysis with landmarks**			
**HBeAg-seroclearance within 24 months from initiation of antivirals**			
	n	HR (95% CI)	*P*
HBeAg-loss	261	0.57 (0.21–1.55)	0.27
HBeAg-maintained	2,121		
**HBeAg-seroclearance within 36 months from initiation of antivirals**			
	n	HR (95% CI)	*P*
HBeAg-loss	434	0.89 (0.49–1.63)	0.72
HBeAg-maintained	1,948		
**HBeAg-seroclearance within 48 months from initiation of antivirals**			
	n	HR (95% CI)	*P*
HBeAg-loss	599	1.02 (0.57–1.82)	0.95
HBeAg-maintained	1,783		
**HBeAg-seroclearance within 60 months from initiation of antivirals**			
	n	HR (95% CI)	*P*
HBeAg-loss	725	1.10 (0.63–1.93)	0.73
HBeAg-maintained	1,657		

HCC, hepatocellular carcinoma; IPTW, inverse probability of treatment weighting; HR, hazard ratio; CI, confidence interval; HBeAg, hepatitis B virus envelope antigen

Cox proportional hazard regression analysis, time-dependent Cox regression analysis, and time-dependent Cox regression analysis with landmarks were performed. The results were expressed in terms of HR, 95% CI, and *P* values.

Table S9. Baseline characteristics of the Delayed-suppression and Early-suppression groups before and after inverse probability of treatment weighting.

Characteristics		Before IPTW DNA suppression within 4 months				After IPTW DNA suppression within 4 months			
	n = 1,407	Delayed-suppression *n* = 694	Early-suppression *n* = 713	*P*	SMD	Delayed-suppression *n* = 694	Early-suppression *n* = 713	*P*	SMD
Age, years	42 (33–51)	44 (33–52)	42 (34–50)	0.480	0.032	42 (33–51)	42 (34–50)	0.98	0.007
Sex, male, %	851 (60.5%)	422 (60.8%)	429 (60.2%)	0.83	0.013	406 (58.5%)	429 (60.2%)	0.61	0.033
Antiviral, %									
Entecavir	799 (56.8%)	397 (57.2%)	402 (56.4%)	0.79	0.017	390 (60.2%)	402 (56.4%)	0.97	0.003
TDF	608 (43.2%)	297 (42.8%)	311 (43.6%)			304 (39.8%)	311 (43.6%)		
Platelet count, 10^3/μL	187 (144–227)	191 (152–230)	179 (137–226)	0.01	0.120	184 (146–227)	179 (137–226)	0.36	0.033
Albumin, g/dL	4.1 (3.8–4.4)	4.1 (3.9–4.4)	4.1 (3.8–4.3)	0.03	0.080	4.1 (3.8–4.4)	4.1 (3.8–4.3)	0.54	0.015
AST, U/L	107 (70–211)	96 (64–170)	129 (75–249)	<0.001	0.207	133 (80–255)	129 (75–249)	0.47	0.005
ALT, U/L	118 (78–248)	103 (73–193)	144 (82–293)	<0.001	0.198	143 (83–299)	144 (82–292)	0.99	0.032
HBV DNA, log_{10} U/mL	8.0 (6.7–8.2)	8.0 (7.5–8.2)	7.5 (6.1–8.2)	<0.001	0.558	7.6 (6.3–8.2)	7.5 (6.1–8.2)	0.18	0.124
Total bilirubin, mg/dL	0.9 (0.7–1.2)	0.9 (0.7–1.2)	0.9 (0.7–1.3)	0.01	0.070	0.9 (0.7–1.3)	0.9 (0.7–1.3)	0.71	0.015
Creatinine, mg/dL	0.9 (0.7–1.0)	0.9 (0.7–1.0)	0.9 (0.7–1.0)	0.63	0.007	0.9 (0.7–1.0)	0.9 (0.7–1.0)	0.63	0.023
FIB-4	2.4 (1.4–4.1)	2.3 (1.3–3.8)	2.7 (1.5–4.4)	<0.001	0.191	2.7 (1.6–4.6)	2.7 (1.5–4.3)	0.61	0.008
mPAGE-B	9 (7–11)	9 (7–11)	9 (7–12)	0.62	0.030	9 (7–11)	9 (7–12)	0.69	0.024

IPTW, inverse probability of treatment weighting; SMD, standardized mean difference; TDF, tenofovir disoproxil fumarate; AST, aspartate aminotransferase; ALT, alanine aminotransferase; FIB-4, Fibrosis-4; mPAGE-B, modified PAGE-B

All variables were compared between the HBeAg-loss and HBeAg-maintained groups before and after IPTW. Sex, and the type of antiviral treatment were analyzed by Fisher's exact test. Other variables were analyzed by Kruskal–Wallis test and expressed in median (IQR).
